# Positive Evolutionary Selection of an HD Motif on Alzheimer Precursor Protein Orthologues Suggests a Functional Role

**DOI:** 10.1371/journal.pcbi.1002356

**Published:** 2012-02-02

**Authors:** István Miklós, Zoltán Zádori

**Affiliations:** 1 Rényi Institute, Budapest, Hungary; 2 Veterinary Medical Research Institute, Hungarian Academy of Sciences, Budapest, Hungary; National Cancer Institute, United States of America and Tel Aviv University, Israel

## Abstract

HD amino acid duplex has been found in the active center of many different enzymes. The dyad plays remarkably different roles in their catalytic processes that usually involve metal coordination. An HD motif is positioned directly on the amyloid beta fragment (Aβ) and on the carboxy-terminal region of the extracellular domain (CAED) of the human amyloid precursor protein (APP) and a taxonomically well defined group of APP orthologues (APPOs). In human Aβ HD is part of a presumed, RGD-like integrin-binding motif RHD; however, neither RHD nor RXD demonstrates reasonable conservation in APPOs. The sequences of CAEDs and the position of the HD are not particularly conserved either, yet we show with a novel statistical method using evolutionary modeling that the presence of HD on CAEDs cannot be the result of neutral evolutionary forces (p<0.0001). The motif is positively selected along the evolutionary process in the majority of APPOs, despite the fact that HD motif is underrepresented in the proteomes of all species of the animal kingdom. Position migration can be explained by high probability occurrence of multiple copies of HD on intermediate sequences, from which only one is kept by selective evolutionary forces, in a similar way as in the case of the “transcription binding site turnover.” CAED of all APP orthologues and homologues are predicted to bind metal ions including Amyloid-like protein 1 (APLP1) and Amyloid-like protein 2 (APLP2). Our results suggest that HDs on the CAEDs are most probably key components of metal-binding domains, which facilitate and/or regulate inter- or intra-molecular interactions in a metal ion-dependent or metal ion concentration-dependent manner. The involvement of naturally occurring mutations of HD (Tottori (D7N) and English (H6R) mutations) in early onset Alzheimer's disease gives additional support to our finding that HD has an evolutionary preserved function on APPOs.

## Author Summary

HD amino acid duplex can be found in the active center of different metallo-enzymes. An HD motif is positioned directly on the amyloid beta (Aβ) fragment and on the carboxy-terminal region of the extracellular domain of the human amyloid precursor protein (APP) and a taxonomically well defined group of APP orthologues (APPOs). The conservation of the HD dyad is not position specific and it cannot be seen in a multiple alignment. Yet we show with a novel statistical method using evolutionary modeling that HD motif is positively selected by evolution on APPOs, despite the fact that HD dyad is underrepresented in the proteomes of all species of the animal kingdom. CAED of all APP orthologues and homologues are predicted to bind metal ions including Amyloid-like protein 1 (APLP1) and Amyloid-like protein 2 (APLP2). Our results suggest that HDs on the APPOs are most probably key components of metal-binding domains, which facilitate and/or regulate inter- or intra-molecular interactions in a metal ion-dependent or metal ion concentration-dependent manner. The involvement of naturally occurring mutations of HD (Tottori (D7N) and English (H6R)) in early onset Alzheimer's disease gives additional support to our finding that HD has an evolutionary preserved function on APPOs.

## Introduction

Human Alzheimer precursor protein (APP) gene was brought to the forefront of scientific interest in the late 80's when protein sequencing of the major component of the amyloid plaques, the amyloid β peptide (Aβ) implicated APP in the development of Alzheimer's disease (AD) [1]. Subsequent genetic studies revealed that mutations in or multiplications of the APP gene alone can cause early-onset AD with cerebral amyloid angiopathy [2]. APP has two homologues, *APLP1* and *APLP2* in vertebrates and orthologues, like Appl (*Drosophila*), and apl-1 (*Caenorhabditis*), all over the animal kingdom. All orthologues and homologues show high sequence and domain homology but the Aβ domain remains a unique feature of the vertebrate APPs [3], [4].

APP is a Type-I transmembrane protein with a complex domain organization. So far eight domains were identified on the mammalian APPs, the growth factor like domain, the copper-binding domain, Kunitz-type protease inhibitor domain, the OX2 domain, the glycosylated E2 domain, the unstructured carboxy terminal region of the APP extracellular portion, the transmembrane domain and the short cytoplasmic tail that is involved in transcriptional signaling [5]. Despite intense industrial and academic interest, the physiological and developmental role of APP and the contribution of the different domains to the APP's function are not completely understood.

The human APP gene is ubiquitously expressed not only in glial and neuronal cells but also in almost all tissues that have been examined. The pre-mRNA contains 19 exons and it is alternatively spliced to produce several isoforms. In the brain APP695 is the major component, which compared to the longer versions, is missing the KPI and the OX2 domains on its extracellular portion [6].

**Figure 1:**
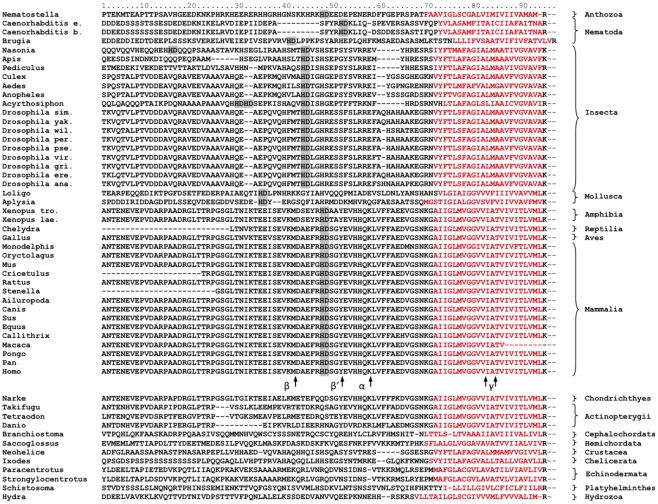
Multiple alignment of the membrane-proximal regions of CAEDs and the transmembrane helices of the APPOs. The predicted transmembrane domains are in red. From the CAEDs only the regions homologous to the predicted metal-binding site of the human APP are shown. The HD dyads are highlighted by gray. Digestion sites of the human α-, β-, and γ-secretases are marked by arrows.

APP is localized to many membranous compartments within the cells. It travels through the endoplasmic reticulum and the Golgi apparatus to reach the cell membrane where it is re-internalized in the lysosomes. During this journey the majority of APP is processed on the cell surface by α-secretases which results in the membrane-bound C83 fragment and the soluble APPsa fragment. C83 is cleaved further in its transmembrane region by gamma secretases and leads to the release of the P3 fragments (Aβ_17–40/42_) and the APP intracellular domain (AICD) into the intercellular space and the cytosol, respectively. However, the minority of the APP might be processed on the amyloid pathway by the β secretase complex which generates APPsb and a 16 amino-acid longer version of C83, the so-called C99 fragment and to a lesser extent it can also cleave within the Aβ domain between Tyr10 and Glu11. The cleavage of the β secretase-generated fragments by γ secretase leads to the release of the AICD into the intracellular compartment and to the generation of Aβ_1–40_, the more neurotoxic Aβ_1–42_, and Aβ_11–40/42_ (see Figure 1) [7], [8], [2].

Several hypotheses sprung up to explain Aβ's contribution to the etiology of AD, including the amyloid cascade [9], the oxidative stress [10], and the signal transduction hypothesis [11], [12]. A Recent model couples Aβ with the loss of ionic homeostasis and the hyperphosphorylation of the Tau protein [13]. Novel experimental data provided additional support to this hypothesis [14]. It seems that all amyloid versions including the P3 fragments are able to oligomerize and the insertion of these oligomers into cellular membranes, by ion channel formation, leads to the perturbation of ionic homeostasis and consequently neurite degeneration and cell death [15], [16], [17], [18].

An HD amino acid duplex has been found in the active center of many different enzymes. Most of these are metalloenzymes like phospholipase A2 [19], metal-dependent phosphohydrolases [20] and metalloproteinases [21]. However, the HD dyad is not restricted to various hydrolyses but it is also an intrinsic part of the active center of the bacterial cycJ/ccmE heme chaperones which are key players in the bacterial cytochrome C biogenesis [22]. In these divergent groups of enzymes HD plays remarkably different roles in the catalytic processes. In sPLA2, H48 (H of HD) functions as a general base and it is assisted by a distal aspartic acid D99 to deprotonate a catalytic water molecule that hydrolyzes the phospholipid ester. The adjacent D49 (D of HD), via its β-carboxyl group coordinates the catalytic Ca2+ cofactor involved in the stabilization of the transition state [23], [24], [25]. In metal-dependent phosphohydrolases HD coordinates the catalytic metal ion by their side chains as intrinsic part of a metal-binding motif H…HD…D. Mutations of HD eliminate or greatly reduce the enzyme activity as it was shown in the case of the YfbR 5-deoxyribonucleotidase [26] and the tRNA nucleotidyltransferase [27] of *E. coli*.

In α-secretases, which are members of the adamalysin/ADAM metalloproteinase family, the fully conserved Asp-416 is involved in intramolecular hydrogen bond interactions and directly follows the last hystidine of the zinc-binding consensus motif HEXXHXXGXXH [28].

In cycJ/ccmE proteins H of the conserved HD covalently binds and releases the hem prosthetic group [29]. The mechanism of binding and releasing of hem is not clear but it depends on the interaction of cycJ/ccmE with other protein components of the cytochrome C maturation pathway.

We have identified an HD dyad on the Aβ domain of the mammalian APP proteins. Although position specific conservation is not observed, we show with a novel statistical method using evolutionary modeling that the motif is positively selected along the evolutionary process in the majority of the APP orthologues (APPOs) despite the fact that no other sequence conservation can be recognized on the carboxy-terminal region of the APPOs extracellular domains (CAEDs). In addition, we also show that HD dyads in the proteome of various organisms are under-represented, which further supports the hypothesis that the prevalence of HD in CAEDs is the result of evolutionary selection rather than arbitrary events. The conservation of HD in CAEDs strongly suggests a functional role of this motif, which most likely involves metal coordination or chelation.

## Materials and Methods

### Proving positive selection of HD motifs

APP orthologues have been collected from the NCBI protein databank with the Blast-P program. The following proteins have been found: Acyrthosiphon pisum, XP_001947569.1; Culex quinquefasciatus, XP_001864483.1; Brugia malayi, XP_001899252.1; Caenorhabditis briggsae, XP_002644641.1; Loligo pealei, ABI84193.2; Aplysia californica, AAT07668.3; Aedes aegypti, EAT42567.1; Drosophila simulans, EDX16764.1; Drosophila yakuba, EDX00795.1; Anopheles gambiae str. PEST, XP_312126.4; Nematostella vectensis, EDO45291.1; Drosophila willistoni, XP_002067462.1; Drosophila persimilis, XP_002027785.1; Drosophila pseudoobscura pseudoobscura, XP_001354498.2; Drosophila virilis, XP_002055698.1; Drosophila grimshawi, XP_001992447.1; Drosophila erecta, XP_001982404.1; Drosophila ananassae, XP_001966309.1; Nasonia vitripennis, XP_001601635.1; Culex quinquefasciatus, XP_001864483.1; Pediculus humanus corporis, XP_002426948.1; Manduca sexta, AAY25024.2; Rattus norvegicus, NP_062161.1; Mus musculus, Q53ZT3; Monodelphis domestica, XP_001373948.1; Equus caballus, XP_001499900.2; Sus scrofa, ABB82034.1; Gallus gallus, AAG00594.1 Canis lupus familiaris, AAX81908.1; Macaca fascicularis, BAD51938.1; Ailuropoda melanoleuca, XP_002920108.1; Oryctolagus cuniculus, XP_002716819.1; Pan troglodytes, AAV74286.1; Callithrix jacchus, XP_002761374.1; Stenella coeruleoalba, AAX81912.1; Xenopus (Silurana) tropicalis, AAH75266.1; Xenopus laevis, AAH70668.1; Pongo abelii, NP_001127014.1; Cricetulus griseus, AAB86608.1; Chelydra serpentina serpentina, AAN04908.1; Apis mellifera, XP_624124.3; Ixodes scapularis, XP_002400744.1; Schistosoma mansoni, CAZ32701.1; Hydra magnipapillata, XP_002154415.1; Neohelice granulata, ACO59955.1; Paracentrotus lividus, CN53783.1; Strongylocentrotus purpuratus, XP_790315.2; Saccoglossus kowalevskii, P_002741027.1; Branchiostoma floridae, XP_002613121.1; Narke japonica, BAA24230.1; Takifugu rubripes, O93279.1; Tetraodon fluviatilis, O73683.1; Danio rerio, NP_690842.1; Tetraodon nigroviridis, CAG05838.1.

**Figure 2:**
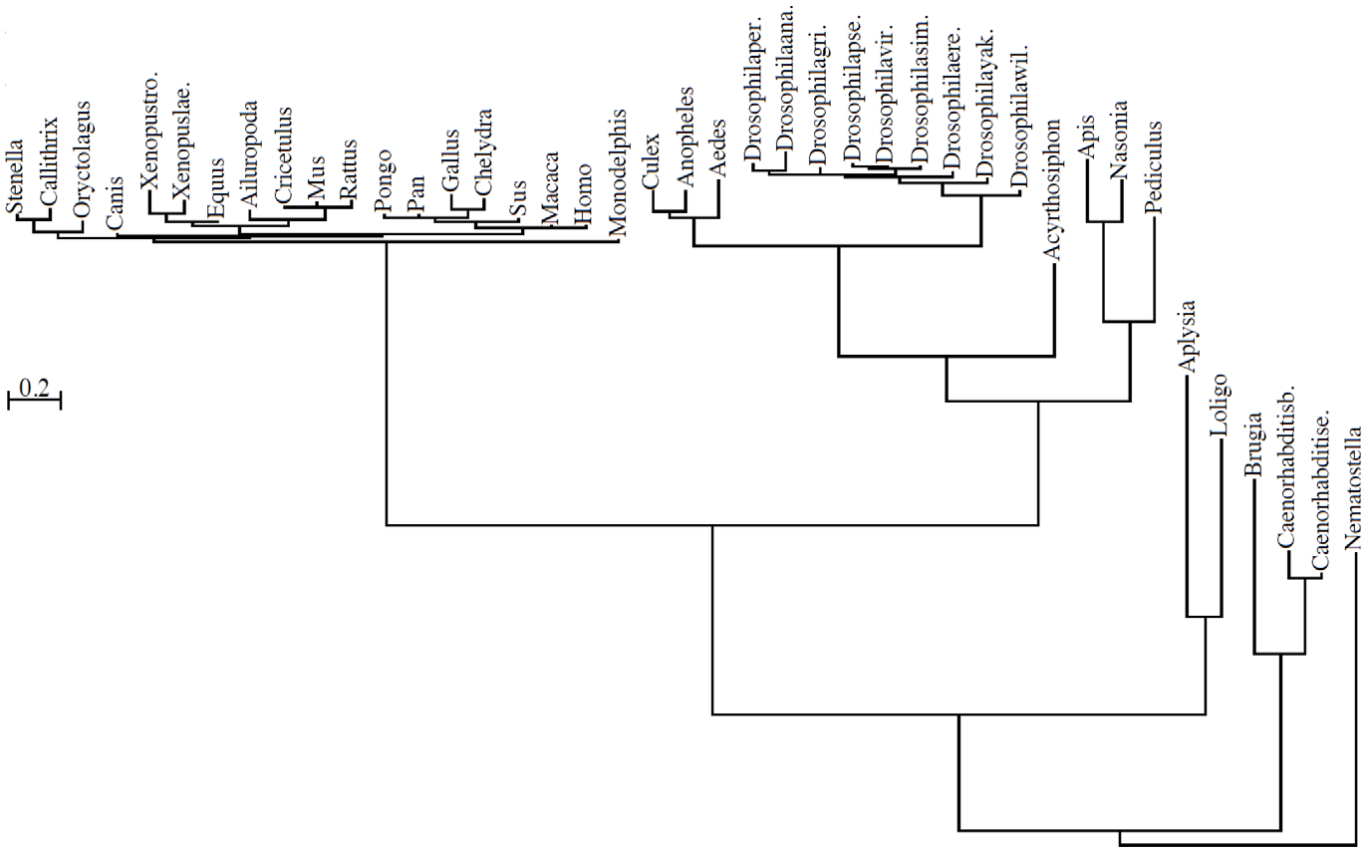
Consensus tree of the membrane-proximal regions of CAEDs containing HD motifs. The tree was calculated from region 1–70 of the alignment shown in Figure 1 by SplitsTree 4.0 using the consensus network method. Bar represents 0,2 PAM distance.

The retrieved proteins were globally aligned with MultAlin [30]. The transmembrane helices of the proteins have been identified by HMMTOP [31] and regions comprising the last 70 amino acids of the CAED sequences and the membrane spanning helices were realigned again with MultAlin. Different regions of this alignment (Figure 1) were used as the inputs for MrBayes 3 [32], a program that samples evolutionary trees from a Bayesian distribution with a Markov chain Monte Carlo method. We used the default parameters of MrBayes, and the convergence of the chain was checked by the log-likelihood trace. We discarded the first half of the chain as burn-in and the consensus tree of the sampled trees was obtained by the consensus network method [33] using SplitsTree 4.0 [34]. There were no ambiguities in the topologies of the trees. As an example, a tree is shown in Figure 2, created from the alignment corresponding to the last 70 amino acids of human CAED.

We implemented a program in the Java 1.6 language that takes an evolutionary tree and a sequence labeling its root, and evolves the sequences on the tree according to a substitution model represented with a continuous time Markov model. The Markov model is given by its rate matrix, *Q*, we used the rate matrix being equivalent with the BLOSUM62 matrix, see [35] for details. The exponent of the matrix contains the so-called transition probabilities. For example, the entry in row *k* and column *l* contains the probability that a site is in a particular amino acid *a_k_* after evolutionary time *t*, given that the site was in amino acid *a_l_* in the beginning. The exponet of the matrix by definition is
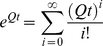
(1)but for technical reasons, we use the diagonalized form of the matrix. If
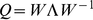
(2)where *Λ* is a diagonal matrix containing the eigenvalues of *Q*, then
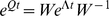
(3)which is much easier to calculate since
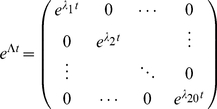
(4)More details about the mathematical background of the calculation method are given elsewhere [36]–[38].

Each site in the sequence is evolved independently of the other sites, and the evolution on each branch of the evolutionary tree is also independent of the evolution on other branches. We took the evolutionary tree generated by SplitsTree 4.0, put the *Nematostella vectensis* CAED to the root, and let the sequences evolve according to a substitution model being equivalent to the BLOSUM 62 score matrix. Namely, for each site, and each edge of the tree from the root towards the leaves, we took the amino acid at the incoming vertex of the edge. We calculated the exponent of the rate matrix using the evolutionary time assigned to the edge in question, then generated a random amino acid for the outgoing vertex of the edge. If the amino acid at the incoming vertex was *a_l_*, than the amino acid at the outgoing vertex of the edge was generated from the distribution taken from the *l*th column of the exponentiated rate matrix. We ran the program simulating the evolution on the tree 10 000 times, and 10 000 sequence sets containing the sequences generated at the leaves of the tree were collected.

The number of every amino acid dyad in the generated sequences was counted, and the distribution of the sequence sets with different number of sequences containing any given dyads was calculated. The observed number of CAEDs with HD dyads (41 altogether) was compared with the empirical distribution of the 10 000 sequence sets calculated to the HD dyad to test the following hypothesis:

**H_0_.** The amino acids in the CAEDs evolved neutrally; there is no selection force to maintain at least one HD motif in the domain.

**H_1_.** The amino acids in the CAEDs did not evolve neutrally; there is a selection force to maintain at least one HD motif in the domain.

The *p*-value is the probability of a value at least as high as the observed value (41 HD containing “real” sequences) assuming the *H*_0_ hypothesis. The neutral evolution hypothesis can be tested for any dyad, and thus, similar *p*-values were calculated for other dyads, as well.

### Inferring the HD diamino-acid distribution in proteomes

We downloaded the Uniprot/Swisprot database, 2011-05-31 release (ftp://ftp.ebi.ac.uk/pub/databases/uniprot/knowledgebase/) in fasta file format. Derivative databases were generated for all species with more than a thousand deposited sequences. For each database, the empirical distribution of single amino acids as well as the empirical distribution of diamino-acids was calculated. From this, the log-odds were calculated for each pair of amino acids. By definition, the log-odds for amino acid pairs *a* and *b* is:
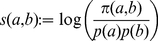
(5)where p(a) and p(b) are respectively the probabilities of amino acid *a* and *b* in the single amino acid distribution, and *π*(*a*,*b*) is the probability of the *ab* diamino-acid motif in the diamino-acid distribution. (See Table S1).

### Prediction programs

For secondary structure prediction the PSIPRED [39] and Jpred [40] software were applied. PSIPRED uses position specific scoring matrices generated by PSI-BLAST to predict protein secondary structure by a two-stage neural network. The program proved to be the most accurate (76.5% to 78.3%) among all investigated methods in the third Critical Assessment of Techniques for Protein Structure Prediction experiment (CASP3) [39].

Jpred runs also a neural network predictor Jnet v3.0, which combines PSI-BLAST position scoring matrix with hidden Markov profiles and achieved a secondary structure prediction score of 81.5% in blind experiments. In a validation test the two programs produced largely overlapping results, however on a portion of the data set one or the other programs gave more accurate prediction [40], so combined application of the two programs and comparison of their results might sometimes be beneficial.

Metal-binding was predicted by the SVMProt server [41] and the MetalloPred program [42].

MetalloPred classifies proteins from sequence derived features (like amino acid composition physicochemical properties and pseudo-amino acid composition) by using a three level cascade of neural networks. The 1st layer of the cascade is for finding metalloproteins, the 2nd layer for the main functional classes (e.g transition metal); and the 3rd layer for identification of the bound metal (e.g. zinc). The accuracy of the program at the first level is reported to be >80%, while the overall accuracy for the correct metal recognition is higher than 60%.

The SVMProt server runs support vector machine prediction systems to predict metal-binding proteins with 10 metal-binding classes (e.g. sodium-binding and zinc-binding, etc). It recognized metal-binding domains and multi-domain metal-binding proteins with more than 80% accuracy in validation tests.

## Results

### Alignment and sequence similarity

Screening of protein databases revealed an HD dyad on the mammalian APP proteins. The motif is positioned directly on the amyloid beta fragment of human APP (amyloid beta conventional numbering H6, D7; APP conventional numbering H677, D678) and it seems to be conserved among not only mammals but four legged vertebrates (tetrapoda), too. No APLP1 or APLP2 orthologues of any animals contain the motif in their functionally homologous region (data not shown).

To clarify the functional and evolutionary significance of the HD motif in APP proteins, first the available APPOs were collected from protein databanks and their CAEDs and the transmembrane (TD) domains were aligned (Figure 1). The alignment revealed that the presence of Aβ domain, embedded in the CAED and TD, is indeed restricted to vertebrate APPs as it was earlier recognized by others [3], [43]. The uniqueness of Aβ in vertebrate APPs is the direct consequence of the fact that the CAEDs and TDs show little sequence conservation between taxonomically divided larger groups like insects, vertebrates and nematodes. In-group conservation of CAED inversely correlates with diversity, e.g. the CAED of insects are less conserved than the CAED of vertebrates. So, in general it can be stated that the more related the animals, the more similar their CAED and TD are on the APPOs. However, there are some exceptions. Interestingly, the CAED and Aβ of the cartilaginous fish *Narke Japonica* [44] show significantly higher homology to human APP than to that of the more related bony fishes [45].

### Motifs and secondary structure predictions

Despite the lack of conservation in the animal kingdom, the majority of the CAEDs contain an HD dyad in their last 70 amino acid region (CAED_C70_). In spite of intensive search we were unable to find any local conservation around the dyad or a conserved amino acid pattern in the CAEDs, which would include the HD motif. In human Aβ HD is part of a presumed RGD-like integrin-binding motif RHDS [46], [47] which was shown to promote cell adhesion and α5βl integrin binding of the soluble form Aβ but not of APP or the fibrillar form of Aβ [48]. The selectivity of integrin binding toward soluble Aβ could be explained with the structural requirements of RGD binding. RGD must be on a tongue-like loop to fit into the ‘well-shaped’ ligand-binding pocket of the integrin where the carboxilate of the aspartic acid coordinating a zinc ion and the basic arginine moiety through a salt bridge anchors the ligand [49], [50], [51]. It seems plausible that RHD of the soluble Aβ can fold into loop while RHD of the more structured APP and the fibrillar form cannot. It also follows from the mechanism that HD, with high probability, cannot be evolutionary preserved on CAEDs to promote integrin binding (or at least not in an RGD-like manner) because the arginine residue in the RHD motif of the human Aβ does not demonstrate reasonable conservation, and it is not conserved even among mammals (GHD in rodents). In fact, the RXD motif is missing from the majority of non-vertebrate CAEDs.

Dissimilar sequences frequently share similar secondary structures and folds [52], so we have investigated with secondary structure prediction programs whether CAEDs and especially the HD surrounding areas share any common secondary structures. Aβ has a propensity for conformational change; its actual structure largely depends on the interacting environment. Therefore it can be considered as a so called “dual personality fragment” [53] (an extended concept of “chameleon sequence” [54], [55]). The monomeric Aβ is unordered in aqueous solution and takes up a helical structure in membrane-mimicking media [56]–[61]. By contrast, in amyloid fibrils and ion channel-forming aggregates residues 18–42 adopt a β-strand–turn–β-strand motif [62], [18]. Residues 16–23 compose a discordant helix which seems to be critical for fibril formation [63]. Contrary to the Aβ, not much is known about the CAEDs structure in APPOs except that human CAED was predicted to be unordered [5]. Our prediction results have been inconclusive. Human CAED_C70_ is predicted to be largely unordered coil, in which the HD is bracketed by a short helix and a β strand. This prediction does not exactly match any Aβ experimental data, which is not surprising, considering that predicting secondary structure of dual personality fragments is intrinsically difficult. The divergent CAEDs did not show uniformity in secondary structural features either on the full length CAEDs or in the HD surrounding areas, though in the CAEDs of vertebrates and some insects HD is located at the carboxy-terminal of a (5–10 amino acid length) helix (Figure S1).

### Taxonomic segregation of CAEDs with HD motif

The presence of HD on different APPOs is far from being arbitrary and shows a progressive taxonomic distribution in certain animal groups. Though it appears first in the Anthozoa class of the Cnidaria phylum, it is absent from the APPOs of the Hydrozoa class, the Platyhelminthes and Echinodermata phyla. Besides tetrapods, it can be found in all the available APPOs of insects, mollusks and nematodes, and it is completely missing from the members of the related primordial taxons of the deuterostomia and arthropoda lineages like cephalochordates, bony and cartilaginous fishes, crustaceans and chelicerate arthropods (Figure 1). The evolution of tetrapods from sarcopterygian fish could be dated in the Late Devonian period [64] while insects were separated from other arthropods around 400 million years ago in the early Devonian, more than 150 million years later than crustaceans and chelicerate arthropods [65], [66]. Based on these data it is tempting to speculate that the HD dyad independently evolved in the naïve ancestors of tetrapods and insects and it is sustained in the CAEDs of the species of these and other taxons by a similar if not identical evolutionary force. However, the division of CAEDs regarding the presence of HD and the lack of recognizable sequential or structural conservation in CAEDs around the HD dyad forced us to investigate the possibility that the appearance of these amino acids in the APPs of different animals would be the result of random or arbitrary evolutionary events.

### The HD motif is underrepresented in the proteomes, but overrepresented in the CAED domains

First we examined whether the frequent occurrence of the HD on CAEDs could be the result of overrepresentation of HD in the biota and most importantly in the animal proteomes.

**Figure 3:**
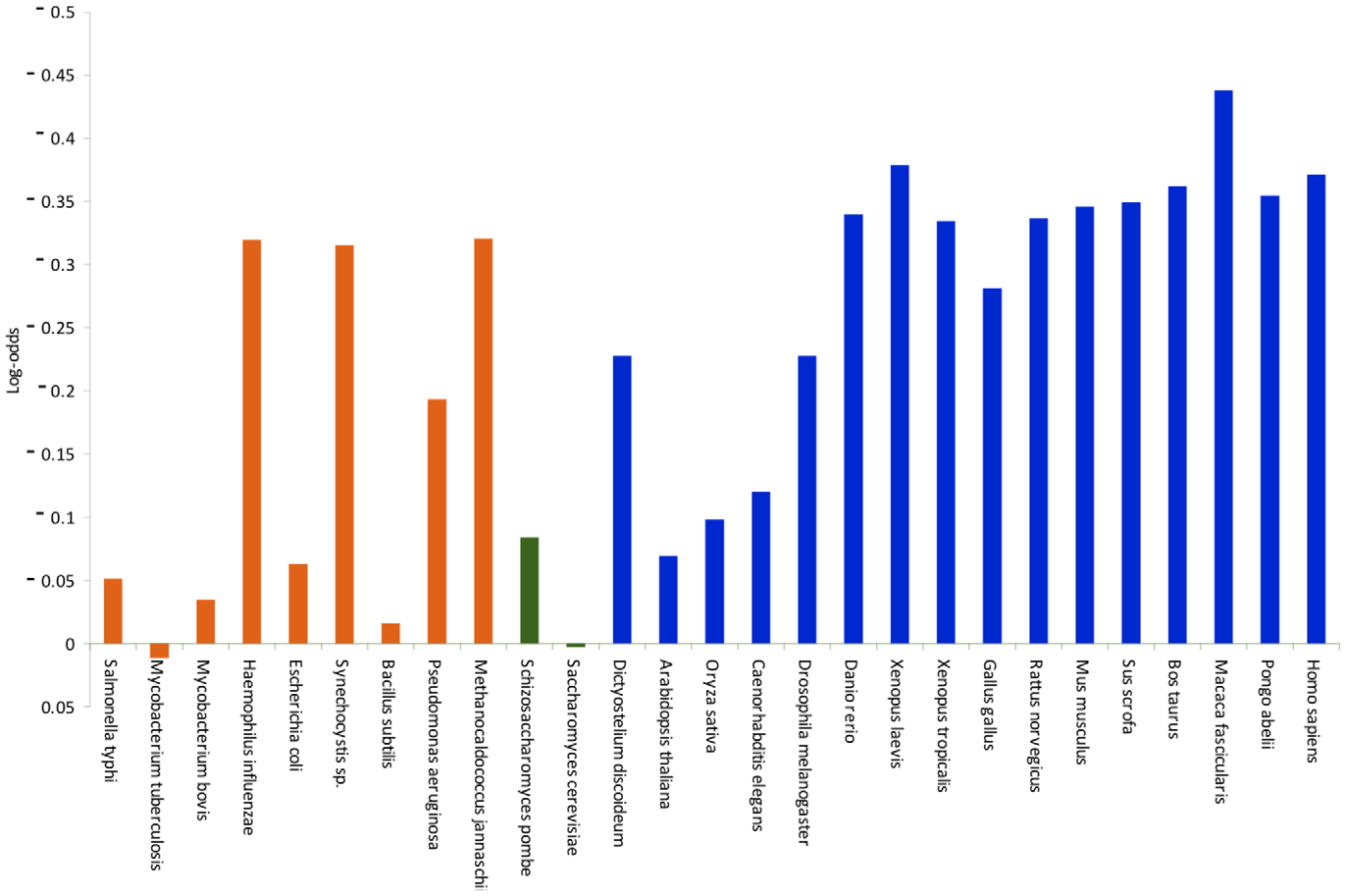
The log-odds values of the HD dyad in the proteomes of several organisms from the biota. Orange, green and blue columns represent prokaryotes, single-cell eukaryotic and multicell eukaryotic organisms respectively.

The log-odds of the HD motif as defined in Equation (5) in the vast majority of investigated proteomes resulted in negative values. This indicates that the HD motif is underrepresented in the biota in general, as well as in specific species. Among single-cell organisms HD frequency fluctuates and the log-odds can even take positive values, while in multi-cell organisms, regardless of their taxonomical place, it always remains negative. Based on the presently available data, there seems to be an inverse correlation between the log-odds and the evolutionary development of the phyla in the animal kingdom. In the investigated species the values decrease from primitive Bilateria towards modern Bilateria, and they reach the minimum in vertebrates (Figure 3). It is worthwhile mentioning that in the human proteome the number of HDs lags farthest behind the expected value calculated by the amino acid composition indicating that HD has the smallest log-odds among all amino acid dyads (Table S1).

On the other hand, the log-odds value of the HD motif in the CAED_C70_s is 1.355. Namely the HD motif is overrepresented in the CAED_C70_s, its occurrence is much more frequent than the independent distribution of amino acids would indicate.

### Positive selection of HD motifs

We have also developed a computer program to study whether neutral evolutionary forces are able to produce such overrepresentation of HDs on the CAEDs. The program takes an evolutionary tree (e.g. the tree is shown in Figure 2) and a sequence labeling its root, and evolves the sequence at the root on the tree by keeping the original distances among the leaves according to a substitution model (a continuous time Markov model) representing neutral evolution. After a user defined number of iteration of the neutral evolutionary process, the program calculates the number of any amino acid dyads (e.g. number of HD) and the number of sequences containing a given dyad (e.g. 26 HD can be on 22 sequences if some of the sequences contain more than one HD) in every set of the computationally evolved sequences.

As an input tree, first the evolutionary tree of the CAED_C70_s was chosen (generated from region 1–70 of the alignment (see Figure 1)) by taking the *Nematostella* sequence as a root. We have chosen the sequence of the simplest organism to evolve because this way the direction of the evolution in the simulated processes gives the closest approximation of the reality.

**Figure 4:**
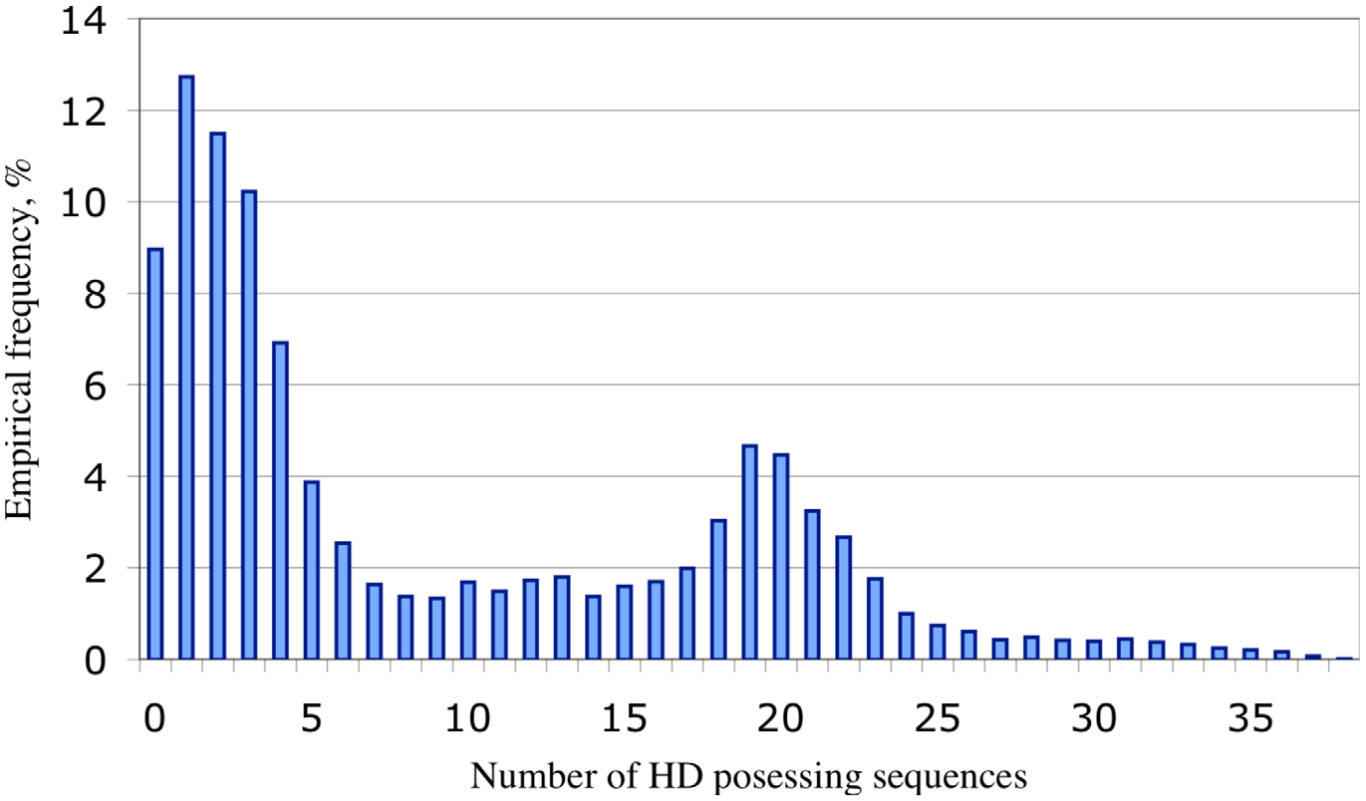
The empirical H_0_ distribution of 10 000 sequence sets assuming neutral evolution. Every column represents a group of sequence sets with a certain number (0–41) of HD containing sequences. Numbers on the X axis correspond to the number of HD containing sequences in the group. Groups with 39–41 HD containing sequences have 0 member and they are not indicated on the X axis. Labels on the Y axis show the size of the groups in percentage of the total 10 000 sets.

As shown in Figure 4, after 10 000 iterations the distribution of the evolved sequence sets with different number of HD motif containing sequences is not unimodal. This is due to the correlation amongst the sequences labeling the leaves of the evolutionary tree. Indeed, if a motif is represented in one of the sequences, the probability that it is also represented in its closely related homologues is higher. In more than 33% of the cases (9,12%+12,81%+11,34%) from the 10 000 simulations, the HD dyad occurred only in no more than two evolved sequences (Figure 4) and never occurred in all 41 computationally evolved sequences.

Therefore we conclude that we can reject the neutral evolution hypothesis with a *p* value smaller than 0.0001, and HD is kept in the CAEDs by a selective evolutionary force. The cross-validation showed that three dyads (KM, EP and HQ) amongst the possible 400 ones were significant at *p* = 0.01 level (Table S2 Panel A,). This result is comparable to the expected 4 ones (1% of the 400).

Though a large number of CAED_C70_ were tried as input (among them the *Human* and *Drosophila* sequences) to test the effect of the input sequence on the final outcome, in every case the *p* value of HD dyad remained smaller than 0.0001. Using different trees, generated from different parts of the alignment, can have an influence on the amino acid composition of the evolved sequence sets and consequently on the distribution of the dyads. So we tested several trees derived from shorter and longer regions of the alignment, yet the P value for HD never reached 0.0001, (Table S2 Panel B, and C,) showing that the result is not the consequence of an arbitrary choice.

The lack of position-specific conservation raises the question of how the HD dyad can be kept by a selective evolutionary force without position-specific conservation. In our computer simulations, more than 20% of the simulated sequence sets contained at least one sequence with 2 or more HD dyads, hence, we conclude that the probability for the emergence of a new HD dyad is relatively high. If the evolutionary force is only for maintaining at least one of the HD dyads, then the deletion of the old HD dyad will not be prevented by selection just as it happened in the majority of the modern sequences.

To test further the hypothesis that HD motifs might appear by random mutations, we repeated the simulation of neutral evolution, but now we put the *Hydra magnipapillata* sequence to the root, which does not contain a HD dyad. The distribution of the number of sequences containing a HD motif still followed a multimodal distribution similar in shape to the one on Figure 4, but the distribution was shifted towards smaller values, with an average number of 3.32 HD containing CAEDs/sequence set in contrast to 9.68 HD containing CAEDs/sequence set when we put the HD containing ancestor to the root (data not shown).

### Metal-binding prediction

From a structural point of view, an important recognizable common feature of the CAED_C70_s is that despite the lack of sequence similarity they are rich in metal coordinating amino acids (His, Glu, Gln Asp, Asn, Tyr, Ser, Thr Arg, Lys). Certain combinations of these amino acids like EE ED and DD are also relatively frequent on CAED_C70_s (they occur on 34, 37 and 17 protein segments of the 53 CAED respectively), though their occurrences stay below that of the HD. These dyads are also able to coordinate metal ions. EE was reported as part of manganese and nickel coordinating motifs [67], [68]. ED and DD bind calcium [69], [70] magnesium [71], [72] and manganese [73], [74]. Interestingly, other combination of E, D and H amino acids H-7X-H, E-2X-H, H-3X-H, E-4X-D, which can be found in several metal-binding motifs [75] are also frequent (42, 34, 18, 22 occurrence respectively) on CAEDs. These observations prompted us to investigate *in silico* whether the CAEDs could have metal-binding capabilities. Surprisingly, the last 60–70 amino acid of all CAEDs were predicted to be metal-binding domains, by 2 different prediction programs, regardless of the presence or absence of the HD motif (Table S3). Both programs Metallopred and SVMProt achieved more than 80% accuracy in validation tests [41], [42] so the chance that all of these sequentially non-homologous protein segments, with similar functions, would be falsely predicted to have metal-binding capabilities seems to be marginal. Furthermore, the carboxy-terminal region of the extracellular domain of APLP1 and APLP2 orthologues is also predicted to bind metal ions, which suggests that metal-binding capability near the transmembrane anchor are evolutionary maintained and it is indispensable in order that APP orthologues and homologues exert their normal biological functions. This is coincident with the observations that amyloid deposits contain high levels of copper, iron, and zinc [56], [76] and natural Aβ is a metalloprotein [77]. What is more, investigations of the metal-binding properties of the Aβ revealed that both amino acids of HD motif (H6 and D7) are involved in metal coordination. The first sixteen amino acids of human Aβ can bind zinc by inter and intramolecular coordination [78], [79], [80] and H6 contributes to the latter one. Moreover age-related isomerization and racemization of D7 result in zinc dependent oligomerization of Aβ(1–16) peptide and it causes conformational change in the His6–Ser8 region of Aβ(1–16) which retains its zinc-binding capacity by the involvement of L-iso-D7 [56], [81]. Molecular dynamic simulations on Aβ models and EPR studies are also involving unmodified D7 in inter- [82] and intramolecular metal coordination [83] and oligomerization [82].

Taken together, these data indicate that H and D may be involved in metal coordination not only in human Aβ but also on APPOs, and their evolutionary selection can be related to this function.

## Discussion

Investigation of the CAEDs of evolutionary distant animals revealed that despite the low sequence similarity, the majority of them contain an HD dyad and their membrane-proximal 60–70 amino acid regions are predicted to bind metals. The HD-containing CAEDs belong to the species of well-defined taxonomic groups such as tetrapodes, insects, mollusks and nematodes. We have shown using an evolution model system that although HD is negatively selected in the proteome of different animals, the presence of the HD dyad on CAEDs is most likely the result of positive selection. We want to emphasize that the conservation of the HD dyad is not position specific; hence its conservation cannot be seen in a multiple alignment. However, the positive evolutionary selection of HD has been proved by statistical testing of the sequences. Under the neutral evolution hypothesis, namely, assuming no selection force for maintaining the HD dyads, the probability of the observed abundance of the HD dyads is less than 0.0001.

Computer simulations showed that the emergence of an HD dyad in the CAED sequences is likely even under neutral evolution. Two of the CAEDs contain more than one HD dyads. According to the simulation the probability of observing such multiple occurrences in at least one of the modern sequences is over 20% even in the case of neutral evolution. The probability that at least one of the evolving intermediate sequences contained multiple dyads is even higher. Without the selection force, these appearing HD dyads could mutate, thus the sequence could lose at least one, or even all of them. In case of a selection force, at least one of the HD dyads is preserved in the sequence. However, this dyad might be the one that appeared by random mutations, and the older HD dyad might be deleted. This scenario could explain why we see HD dyads in the majority of sequences without site-specific conservation. Similar migration of functional elements have already been described for transcription factor binding sites in DNA promoter regions as the so-called binding site turnover [84], [85], [86]; however, to the best of our knowledge, this is the first time that such motif turnover is described for proteins.

The evolutionary selection of HD strongly suggests a functional role of the motif in APPOs, which is probably related to metal coordination. As the HD motif is part of the extended catalytic network of several enzymes, the contribution of the CAEDs' HD to the formation of a catalytic center through inter- or intramolecular interaction may not be excluded. However, the lack of conservation in the vicinity of HD, the variable position of the motif on the CAEDs and the multiple copy occurrences in certain proteins indicate a low probability for this supposition. The same arguments which oppose enzymatic activity, together with the fact that membrane-proximal region of the CAEDs seem to have metal coordination capabilities, rather suggest that HDs on CAEDs could be key components of metal ion-coordinating domains which facilitate and/or regulate inter- or intramolecular interactions in a metal ion-dependent or metal ion concentration-dependent manner. This notion is supported by the findings that Aβ has metal-binding capability [56], [76]–[83], structural plasticity [57]–[60] metal and metal concentration dependent propensity for structural changes [56], [80], [82] and metal ions (Cu2+ and Zn2+) facilitate the intermolecular contact between Aβ peptides [82], [87] in which H and D are involved by metal coordination [81], [82].

APP and its derivatives interact with large number of proteins. Aβ binds its homologous sequence on APP and also facilitates the oligomerization of the β-secretase cleaved APP C-terminal fragment, C99 [88]. Obviously, mammalian CAEDs directly interact with α- and β-secretases (as they are digested by them) and likely with some member(s) of the γ-secretase complex (all components are membrane proteins and have extracellular parts). Besides the well-studied cytoplasmic carboxy-terminal interacting adaptor proteins [8], [89], [90] large numbers of other extracellularly interacting APP partners were identified with poorly characterized binding features [91]. Both intracellular and extracellular interactions modulate APP processing [92], [93]; consequently, their perturbation could lead to elevated production of neurotoxic Aβ species and the development of AD.

If HD is involved in any molecular interaction, which influences APP processing or has any other function that influences the development of AD, then certain mutations of HD may facilitate the manifestation of AD. In fact, there are reports supporting the biological significance of these amino acids in the development of AD. Naturally occurring mutations of HD are involved in the early onset of familial AD in cases from Japan (D7N) [94] and England (H6R) [95]. These observations provide additional support to the functionality of HD on APPOs.

Though substantial amount of data has already accumulated about the pathogenesis and development of AD, finding the cure may require greater knowledge about the physiological role of APP. We hope that our results can stimulate new investigations and contribute to the better understanding of APP's involvement in the development of AD.

## Supporting Information

Figure S1 Secondary structure predictions of some selected CAED_C70_.
(PDF)

Table S1 The log-odds values of the amino acid dyads in the proteomes of several organisms from the Biota. Table A, shows the number of the amino-acids in the proteomes; Table B, shows the log-odds values which are calculated by equation 5. The first amino acids of the dyads are represented on the vertical axis while the second amino acids are represented on the horizontal axis.
(PDF)

Table S2 The p values of the different amino acid dyads in the neutral evolution simulation. The values of Panels A, B, and C were calculated from different regions (1–70, 10–54 and 1–96, respectively) of the alignment shown in Figure 1. Table A, shows the p values of 10 000 simulated runs. Table B, contains the sum of amino acid dyads in the examined regions of the 41 CAEDs. The first amino acids of the dyads are represented on the vertical axis while the second amino acids are represented on the horizontal axis.
(PDF)

Table S3 Metal-binding prediction results on the CAEDs of APPOs.
(PDF)
