# Supplementary material for: Positive Evolutionary Selection of an HD Motif on Alzheimer Precursor Protein Orthologues Suggests a Functional Role
Source: PLoS Comput Biol. 2012 Feb 2;8(2):e1002356. doi: 10.1371/journal.pcbi.1002356 (PMC3271017; doi:10.1371/journal.pcbi.1002356)
Supplement: Figure S1 — Secondary structure predictions of some selected CAEDC70. (PDF) [file pcbi.1002356.s001.pdf]

A, *Nematostella*

```
JPRED

QUERY            : APTTPSAVHGEEDKNKPHRKHEERERHHGRHGNSKKHRKHDEEDEPENERDFFGEPRSPAT : QUERY
UniRef90_A7RTM2  : APTTPSAVHGEEDKNKPHRKHEERERHHGRHGNSKKHRKHDEEDEPENERDFFGEPRSPAT :
                 : 1---------11--------21--------31--------41--------51--------  :
OrigSeq          : APTTPSAVHGEEDKNKPHRKHEERERHHGRHGNSKKHRKHDEEDEPENERDFFGEPRSPAT : OrigSeq

Jnet             : --------------------HHHHHHH---------------------------------- : Jnet
jhmm             : ------------------------------------------------------------- : jhmm
jpssm            : ------------------HHHHHHHHHH--------------------------------- : jpssm

Lupas 14         : ------------------------------------------------------------- : Lupas 14
Lupas 21         : ------------------------------------------------------------- : Lupas 21
Lupas 28         : ------------------------------------------------------------- : Lupas 28

Jnet_25          : -------B-B-----------------------------------------BB-------- : Jnet_25
Jnet_5           : ------------------------------------------------------------- : Jnet_5
Jnet_0           : ------------------------------------------------------------- : Jnet_0
Jnet Rel         : 9988877777777777650000000000057777765535677777645422114677788 8 : Jnet Rel


PSIPRED

AA: Target sequence
C o n f :  9 9 8 8 8 8 5 1 3 7 6 4 5 7 9 9 6 5 1 2 5 8 8 8 7 5 2 0 6 8 8 9 9 6 2 0 1 4 6 7 8 8 8 8 8 7 6 3 2 2 3 4 5 8 9 9 99 9 9
P r e d :  C C C C C C C C C C C C C C C C C C C H H H H H H H H C C C C C C C C C C C C C C C C C C C C C C C C C C C C C CC C C
    A A :  A P T T P S A V H G E E D K N K P H R K H E E R E R H H G R H G N S K K H R K H D E E D E P E N E R D F F G E P RS P A
```

B, *Caenorhabditis e.*

```
JPRED

QUERY            : SSSSTSSESDEDEDKNIKELRVDIEPIIDEPASFYRHDKLIQSPEVERSASSVFQP : QUERY
UniRef90_Q10651  : --------------KNIKELRVDIEPIIDEPASFYRHDKLIQSPEVERSASSVFQP :

                 : 1---------11--------21--------31--------41--------51---- :
OrigSeq          : SSSSTSSESDEDEDKNIKELRVDIEPIIDEPASFYRHDKLIQSPEVERSASSVFQP : OrigSeq

Jnet             : -----------------HEEEE---------------------------------- : Jnet
jhmm             : ---------------HHEEEEE----------------H----------------- : jhmm
jpssm            : -----------------HHHHH---HHH---------------------------- : jpssm

Lupas 14         : -------------------------------------------------------- : Lupas 14
Lupas 21         : -------------------------------------------------------- : Lupas 21
Lupas 28         : -------------------------------------------------------- : Lupas 28

Jnet_25          : ----------------B--B-B-B--BB---B-BB-B--BB----B---B--BB-- : Jnet_25
Jnet_5           : -----------------------B-------------------------------- : Jnet_5
Jnet_0           : -------------------------------------------------------- : Jnet_0
Jnet Rel         : 99888777765565400000002330001466523345056677665667763478 : Jnet Rel

PSIPRED

C o n f : 9 8 8 8 8 8 8 8 8 5 5 6 6 3 0 4 1 0 0 0 0 0 0 3 2 0 3 3 5 8 8 5 3 1 0 0 0 2 2 4 5 6 9 2 4 4 4 1 2 4 5 5 4 4 7 9
P r e d : C C C C C C C C C C H H H H C C C C H H C C C C C C C C C C C C C C H H C C C C C C C H H H H H C C C C C C C C
    A A : S S S S T S S E S D E D E D K N I K E L R V D I E P I I D E P A S F Y R H D K L I Q S P E V E R S A S S V F Q P
```

C, *Apis*

```
JPRED

QUERY                  : LRTEQAQQEESDSINDNKDIQQQPEQPAAMHSEGLVRAAHSLTHDISHSEPGYSVRREMYHRESRS : QUERY
UniRef90_UPI00015B49D1 : ------------------------------HSEGLIRAAHSMTHDVSHSEPSYSVRREVYHRESR- :
                       : 1---------11--------21--------31--------41--------51--------61---- :
OrigSeq                : LRTEQAQQEESDSINDNKDIQQQPEQPAAMHSEGLVRAAHSLTHDISHSEPGYSVRREMYHRESRS : OrigSeq

Jnet                   : ----HHHHH------------------------HHHHHHHH------------HHHHHHHHH---- : Jnet
jhmm                   : ----HHHHH------------------------HHHHHHH-------------EEEEHHHHH---- : jhmm
jpssm                  : ----HHHHHH-----------------------HHHHHHHHHH----------HHHHHHHHH---- : jpssm

Lupas 14               : ------------------------------------------------------------------ : Lupas 14
Lupas 21               : ------------------------------------------------------------------ : Lupas 21
Lupas 28               : ------------------------------------------------------------------ : Lupas 28

Jnet_25                : -------------B-----B------BB-B-B-BBB-BB--B---B------B-B---BB------ : Jnet_25
Jnet_5                 : ----------------------------------BB-B---B---B-------------------- : Jnet_5
Jnet_0                 : -------------------------------------B---------------------------- : Jnet_0
Jnet Rel               : 997367764046777776543234356654564166665300036788776630000867621799 : Jnet Rel
```

```
PSIPRED

Conf: 9 0 3 8 8 7 7 6 5 4 1 1 3 4 5 7 6 3 2 3 4 7 9 8 7 5 1 2 1 2 1 2 4 5 7 8 8 7 6 6 3 1 2 4 5 7 7 6 8 9 8 7 1 3 5 5 6 5 5
Pred: C C H H H H H H H H H C C C C C C C C C C C C C C C C H H H H H H H H H H H H H H C C C C C C C C C C C C H H H H H H
  AA: L R T E Q A Q Q E E S D S I N D N K D I Q Q Q P E Q P A A M H S E G L V R A A H S L T H D I S H S E P G Y S V R R E M

Conf: 4 2 1 0 2 7 9
Pred: H H H C C C C
  AA: Y H R E S R S
```

D, *Drosophila wil.*

```
JPRED

QUERY           : ATVSSTKVQTALPTVDDDAVQRAVEDVAAAVAHQEAEPQVQHFMTHDLGHRESSFSLRREFAHSHATKEGRN : QUERY
UniRef90_Q0IFC5 : ATISNSPQSTALPTVDDEAVQRAVEEVAAAVAHQEAEPKMQHILSHDTGHSEPTFSVRREIYG--------- : UniRef90_Q0IFC5
UniRef90_Q7QCS9 : -----SPQPTALPTVDDEAVQRAVEEVAAAVAHQEAEPKMQHVLAHDIGHGEP------------------- : UniRef90_Q7QCS9

                : 1---------11--------21--------31--------41--------51--------61--------71 :
OrigSeq         : ATVSSTKVQTALPTVDDDAVQRAVEDVAAAVAHQEAEPQVQHFMTHDLGHRESSFSLRREFAHSHATKEGRN : OrigSeq

Jnet            : ----------------HHHHHHHHHHHHHHHH-------HHHHH----------EHHHHHHH---------- : Jnet
jhmm            : ----------------HHHHHHHHHHHHHHHH-------HHHHHH---------EEEEHHHH---------- : jhmm
jpssm           : --------EEEE-----HHHHHHHHHHHHHHHH----HHHHHHH----------HHHHHHHH---------- : jpssm

Lupas 14        : ------------------------------------------------------------------------ : Lupas 14
Lupas 21        : ------------------------------------------------------------------------ : Lupas 21
Lupas 28        : ------------------------------------------------------------------------ : Lupas 28

Jnet_25         : --B-------BBBBB---BB--BB--BBBBBB---B---B--BB-B-B------B-BB--BB---------- : Jnet_25
Jnet_5          : -----------B--------------B---------------B-------------B--------------- : Jnet_5
Jnet_0          : ------------------------------------------------------------------------ : Jnet_0
Jnet_Rel        : 998887610000455701789999758998720257600554320678888763000076533677777888 : Jnet_Rel


PSIPRED

Conf: 9 4 1 3 6 6 5 2 0 2 4 6 7 6 6 7 6 9 9 9 9 9 9 9 9 9 9 9 9 9 6 4 1 3 1 1 2 1 1 3 5 5 6 5 4 1 2 6 8 8 8 8 7 3 1 3 4 3 5
Pred: C C C C C C C C C C C C C C C C H H H H H H H H H H H H H H H H H H H C C H H H H H H H H H C C C C C C C C H H H H H
  AA: A T V S S T K V Q T A L P T V D D D A V Q R A V E D V A A A V A H Q E A E P Q V Q H F M T H D L G H R E S S F S L R R

Conf: 4 4 2 1 4 5 6 3 2 4 6 9 9
Pred: H H H C C C C C C C C C C
  AA: E F A H S H A T K E G R N
```

E, *Loligo*

```
JPRED

QUERY           : TEARPEQQEDIKTPGFDSETFEDERPAIAEQTIHDLPNHRKKGYIAHVQQQPMIADEVSLDNLYANSHAN : QUERY
UniRef90_Q06BR2 : TEARPEQQEDIKTPGFDSETFEDERPAIAEQTIHDLPNHRKKGYIAHVQQQPMIADEVSLDNLYANSHAN : UniRef90_Q06BR2

                : 1---------11--------21--------31--------41--------51--------61-------- :
OrigSeq         : TEARPEQQEDIKTPGFDSETFEDERPAIAEQTIHDLPNHRKKGYIAHVQQQPMIADEVSLDNLYANSHAN : OrigSeq

Jnet            : ----------------------------HHHHHH---------EEEE----------------------- : Jnet
jhmm            : -----------------------------HHHH----------EEEEE---------------------- : jhmm
jpssm           : ----------------------------HHHHHH-------H-HEE------------------------ : jpssm

Lupas 14        : ---------------------------------------------------------------------- : Lupas 14
Lupas 21        : ---------------------------------------------------------------------- : Lupas 21
Lupas 28        : ---------------------------------------------------------------------- : Lupas 28

Jnet_25         : ----------B----B----B----B-BB--BB--B------BBB--B-----BB--B-B--BBB----- : Jnet_25
Jnet_5          : --------------------------------------------B------------------------- : Jnet_5
Jnet_0          : ---------------------------------------------------------------------- : Jnet_0
Jnet_Rel        : 9987642356777777777765677663066650046665000010005665334676456523567888 : Jnet_Rel


PSIPRED

Conf: 9 8 8 8 2 0 0 0 0 3 6 8 9 9 9 9 8 8 7 4 4 4 3 3 1 1 4 5 6 6 6 5 4 0 5 9 8 8 7 6 4 3 2 0 0 1 0 0 0 3 6 3 0 0 1 0 1 0 1
Pred: C C C C C C H H C C C C C C C C C C C C C C C C C H H H H H H H H H C C C C C C C C C C E E E C C C C C C H H H H C C
  AA: T E A R P E Q Q E D I K T P G F D S E T F E D E R P A I A E Q T I H D L P N H R K K G Y I A H V Q Q Q P M I A D E V S

Conf: 0 0 1 2 1 1 1 3 5 8 9
Pred: C H H H H H C C C C C
  AA: L D N L Y A N S H A N
```

F, *Homo*

JPRED

```
QUERY                  : ANTENEVEPVDARPAADRGLTTRPGSGLTNIKTEEISEVKMDAEFRHDSGYEVHHQKLVFFAEDVGSNKG : QUERY
UniRef90_Q4S0J4        : ANTENHVEPVDARPVPDRGLPTRPGKGVSALKPEEMPQVRMETDERQSAGYEVYHEKLVFFAEDVGSNKG :
UniRef90_Q4S0J4
UniRef90_O73683        : -NTENQVEPVDSRPTFERGVPTRPVTGKS---MEAVPELRMETEDRQSTEYEVHHQKLVFFAEDVGSNKG :
UniRef90_O73683
UniRef90_UPI00016E77F7 : ANTENHVEPVDARPIPDRGLPTRP---------EEMPEVRTETDKRQSAGYEVYHQKLVFFADDVGSNKG :
UniRef90_UPI00016E77F7
UniRef90_Q5XIY5        : ANTDNHVEPVDARPIPERGLPTRP----------EIPKVRLDIEERHNAGYDVRDKRLMFLAEDMGSNKG :
UniRef90_Q5XIY5
UniRef90_Q8UUI8        : -NTHNQVEPVDARPVPDLDLATRPVSGL---KPDDIPELRMEAEERHS---EVYHQKLVFFAEDVSSNKG :
UniRef90_Q8UUI8
UniRef90_UPI00016E7DA8 : ---------------------------VTGKSMEAIPDMRMETEDRQSTEYEVHHQKLVFFAEDVGSNKG :
UniRef90_UPI00016E7DA8
UniRef90_Q3MQ25        : --------------------------GLTNIKTEEISEVKMDAEFRHDSGYEVHHQKLVFFAEDMSSN-- :
UniRef90_Q3MQ25
UniRef90_Q28053        : -----------------------------------ISEVKMDAEFRHDSGYEVHHQKLVFFAEDVGSNKG :
UniRef90_Q28053

                       : 1---------11--------21--------31--------41--------51--------61-------- :
OrigSeq                : ANTENEVEPVDARPAADRGLTTRPGSGLTNIKTEEISEVKMDAEFRHDSGYEVHHQKLVFFAEDVGSNKG : OrigSeq

Jnet                   : ------------------------------------HHHHHH-HH-------EE--EEEEEE-------- : Jnet
jhmm                   : --------------------------------------HH------------EEE-EEEEEE-------- : jhmm
jpssm                  : ------------------------------------HHHHHHHHHH-----EE---EEEEEE-------- : jpssm

Lupas 14               : ---------------------------------------------------------------------- : Lupas 14
Lupas 21               : ---------------------------------------------------------------------- : Lupas 21
Lupas 28               : ---------------------------------------------------------------------- : Lupas 28

Jnet_25                : ------B------------B----B--B-------B--B-B-B-------B-BB---BBBBB--B----- : Jnet_25
Jnet_5                 : -----------------------------------B---------------------BBBB--------- : Jnet_5
Jnet_0                 : ---------------------------------------------------------------------- : Jnet_0
Jnet_Rel               : 9988752567777777777777777777777776510077000000478750100125777524677888 : Jnet_Rel
```

PSIPRED

```
C o n f : 9 8 7 7 7 5 2 0 1 6 7 7 4 5 7 8 9 9 9 8 5 5 7 7 8 8 8 7 5 4 5 5 1 0 0 0 0 0 0 0 0 0 0 0 0 0 2 5 7 7 2 3 5 5 3 1 7 9 8
P r e d : C C C C C C C C C C C C C C C C C C C C C C C C C C C C C C C C C C C C C C C C C C H H H C C C C C C E E E E E E E E
    A A : A N T E N E V E P V D A R P A A D R G L T T R P G S G L T N I K T E E I S E V K M D A E F R H D S G Y E V H H Q K L V

C o n f : 8 6 4 1 0 1 6 8 8 8 9
P r e d : E E E E C C C C C C C
    A A : F F A E D V G S N K G
```

Key for JPRED:

```
Colour code for alignment:
Blue          - Complete identity at a position
Shades of red - The more red a position is, the higher the level of
                conservation of chemical properties of the amino acids
Jnet          - Final secondary structure prediction for query
                H:α-helix; E:β-strand

jalign        - Jnet alignment prediction
jhmm          - Jnet hmm profile prediction
jpssm         - Jnet PSIBLAST pssm profile prediction

Lupas         - Lupas Coil prediction (window size of 14, 21 and 28)

Note on coiled coil predictions  - = less than 50% probability
                                 c = between 50% and 90% probability
                                 C = greater than 90% probability

Jnet_25       - Jnet prediction of burial, less than 25% solvent accesibility
Jnet_5        - Jnet prediction of burial, less than 5% exposure
Jnet_0        - Jnet prediction of burial, 0% exposure
Jnet_Rel      - Jnet reliability of prediction accuracy, ranges from 0 to 9, bigger is better.
```

Key for PSIPRED:

```
Conf: Confidence (0=low, 9=high)
Pred: Predicted secondary structure (H=helix, E=strand, C=coil)
AA: Target sequence
```
