# Supplementary material for: Positive Evolutionary Selection of an HD Motif on Alzheimer Precursor Protein Orthologues Suggests a Functional Role
Source: PLoS Comput Biol. 2012 Feb 2;8(2):e1002356. doi: 10.1371/journal.pcbi.1002356 (PMC3271017; doi:10.1371/journal.pcbi.1002356)
Supplement: Table S1 — The log-odds values of the amino acid dyads in the proteomes of several organisms from the Biota. Table A, shows the number of the amino-acids in the proteomes; Table B, shows the log-odds values which are calculated by equation 5. The first amino acids of the dyads are represented on the vertical axis while the second amino acids are represented on the horizontal axis. (PDF) [file pcbi.1002356.s002.pdf]

	A	R	N	D	C	Q	E	G	H	I	L	K	M	F	P	S	T	W	Y	V		∑	∑ of diads
A	80201	41894	22110	32084	16276	36536	53525	55469	17877	30831	81987	37325	15519	31403	49846	65054	41046	9458	17328	53479		789248	
R	44809	49564	23892	30773	14123	29084	44752	41562	18158	27289	61124	41171	11884	21010	37381	47404	31304	8029	16562	35360		635235	
N	22532	20334	16744	16642	9479	18441	24677	26884	10646	23374	42163	24244	9155	16462	24427	34741	21502	5097	12479	24180		404203	
D	31821	26995	18498	28349	12069	20043	37534	36610	12630	28320	55007	27180	11318	23500	32521	46477	27394	7040	15907	34178		533391	
C	14535	15240	9614	11705	8471	12830	15313	22835	7778	10526	24966	13765	4185	9703	16267	23120	12560	3485	6848	14822		258568	
Q	39920	34279	20582	26071	11180	34093	44456	32741	14986	21896	53332	33131	11296	15233	32272	35020	25694	6132	12503	31346		536163	
E	59432	44803	35038	49209	18499	35117	89577	47134	16769	34547	71495	61651	17496	22554	37606	48457	37832	8033	17365	46396		799010	
G	52672	42501	26562	34824	15421	31755	48877	58831	19460	30614	67970	44551	13449	27448	50866	66255	40783	8839	19490	39752		740920	
H	14881	18336	9647	9682	8334	16038	15079	17845	10522	13720	33846	14756	6131	12474	18940	26559	19396	4048	9101	16668		296003	
I	28135	25994	19945	21774	12551	25327	27623	24100	16811	26763	51575	28367	10183	21488	29178	41404	28743	5867	15544	27484		488856	
L	78295	67801	38975	52350	25243	64966	80235	67983	30945	43946	125506	63560	20290	38388	68558	90532	58453	13225	28912	62888		1121051	
K	45823	36612	26260	34274	14220	28549	56450	35888	15855	30898	56620	51969	14892	19006	36750	43519	34683	6811	17228	38025		644332	
M	22234	11522	9864	13659	4445	10113	20469	14490	5225	9279	22130	15891	5733	8079	12358	17431	12398	2748	6601	15197		239866	
F	22108	22378	15205	18265	10915	20734	21962	24886	11989	20651	47140	19877	7758	18140	22931	39758	23365	5665	13809	23937		411473	
P	56901	39383	20010	31369	13625	32550	51433	62332	16324	21156	60378	31628	10873	22099	70293	64364	35296	8229	18843	42496		709582	
S	60134	51089	30051	42727	21859	44038	57644	64331	25016	35656	92888	44704	15956	34966	67237	106074	50883	12451	23947	54176		935827	
T	42508	27733	19149	26671	15363	26187	39089	42240	15983	26711	60529	29072	11301	24258	41132	52317	35661	8013	15842	43089		602848	
W	9507	8466	6024	7396	2770	6179	9490	8509	3415	6264	13916	9208	3274	5071	6176	10004	7613	2208	3813	8117		137420	
Y	15599	17955	12243	14141	7729	14143	19380	18907	8447	15130	30353	17265	6047	13978	14799	24764	16676	4245	10630	17787		300218	
V	48291	33471	24572	32356	16654	30370	42396	38041	17860	32199	70363	36525	13492	27153	41102	54610	42524	8017	18058	44422		672476	
∑	790338	636350	404985	534321	259226	537093	799961	741618	296696	489770	1123288	645840	220232	412413	710640	937864	603806	137640	300810	673799		11256690	11256690

	A	R	N	D	C	Q	E	G	H	I	L	K	M	F	P	S	T	W	Y	V
A	0.370	-0.063	-0.250	-0.155	-0.110	-0.030	-0.047	0.065	-0.152	-0.108	0.040	-0.193	0.005	0.083	0.000	-0.011	-0.031	-0.020	-0.197	0.124
R	0.005	0.322	0.044	0.020	-0.035	-0.041	-0.009	-0.007	0.081	-0.013	-0.036	0.122	-0.045	-0.102	-0.070	-0.110	-0.085	0.033	-0.025	-0.073
N	-0.231	-0.117	0.141	-0.142	0.018	-0.045	-0.152	0.009	-0.001	0.284	0.044	0.044	0.146	0.106	-0.044	0.031	-0.008	0.031	0.144	-0.001
D	-0.163	-0.111	-0.037	0.113	-0.018	-0.239	-0.010	0.041	-0.107	0.199	0.033	-0.119	0.081	0.184	-0.035	0.045	-0.043	0.076	0.110	0.068
C	-0.222	0.042	0.033	-0.047	0.353	0.039	-0.182	0.293	0.132	-0.067	-0.033	-0.075	-0.190	0.024	-0.003	0.071	-0.099	0.097	-0.009	-0.043
Q	0.059	0.123	0.065	0.024	-0.099	0.287	0.154	-0.076	0.059	-0.063	-0.003	0.074	0.074	-0.254	-0.048	-0.243	-0.113	-0.067	-0.136	-0.024
E	0.058	-0.008	0.198	0.260	0.005	-0.082	0.456	-0.110	-0.228	-0.006	-0.109	0.296	0.113	-0.261	-0.294	-0.318	-0.125	-0.196	-0.207	-0.030
G	0.012	0.015	-0.004	-0.010	-0.101	-0.107	-0.074	0.187	-0.004	-0.052	-0.084	0.047	-0.075	0.011	0.084	0.071	0.026	-0.025	-0.016	-0.109
H	-0.334	0.091	-0.099	-0.372	0.201	0.127	-0.333	-0.089	0.299	0.063	0.136	-0.141	0.057	0.140	0.013	0.074	0.200	0.112	0.140	-0.061
I	-0.199	-0.061	0.126	-0.064	0.109	0.082	-0.229	-0.290	0.266	0.230	0.056	0.011	0.063	0.182	-0.056	0.016	0.092	-0.019	0.174	-0.063
L	-0.005	0.068	-0.034	-0.016	-0.022	0.194	0.007	-0.083	0.046	-0.104	0.115	-0.012	-0.078	-0.068	-0.032	-0.031	-0.028	-0.036	-0.036	-0.065
K	0.013	0.005	0.125	0.114	-0.043	-0.074	0.209	-0.168	-0.069	0.097	-0.127	0.341	0.167	-0.217	-0.102	-0.210	0.004	-0.146	0.001	-0.014
M	0.278	-0.163	0.134	0.182	-0.217	-0.124	0.183	-0.087	-0.191	-0.118	-0.078	0.144	0.200	-0.084	-0.203	-0.137	-0.037	-0.065	0.029	0.057
F	-0.268	-0.039	0.027	-0.067	0.141	0.055	-0.286	-0.086	0.100	0.143	0.138	-0.172	-0.037	0.185	-0.125	0.148	0.057	0.119	0.228	-0.029
P	0.133	-0.018	-0.244	-0.071	-0.182	-0.039	0.020	0.288	-0.136	-0.378	-0.159	-0.252	-0.244	-0.162	0.451	0.085	-0.075	-0.053	-0.006	0.001
S	-0.089	-0.035	-0.114	-0.039	0.014	-0.014	-0.143	0.042	0.014	-0.133	-0.005	-0.183	-0.138	0.020	0.129	0.308	0.014	0.084	-0.043	-0.033
T	0.004	-0.206	-0.125	-0.070	0.101	-0.094	-0.092	0.062	0.006	0.018	0.006	-0.174	-0.043	0.094	0.078	0.041	0.098	0.083	-0.017	0.177
W	-0.015	0.086	0.198	0.126	-0.133	-0.059	-0.029	-0.062	-0.059	0.047	0.015	0.155	0.197	0.007	-0.340	-0.135	0.032	0.273	0.038	-0.013
Y	-0.301	0.056	0.125	-0.008	0.111	-0.013	-0.096	-0.045	0.065	0.147	0.013	0.002	0.029	0.240	-0.247	-0.010	0.035	0.145	0.281	-0.010
V	0.023	-0.127	0.016	0.014	0.073	-0.055	-0.120	-0.152	0.008	0.096	0.047	-0.055	0.025	0.097	-0.032	-0.026	0.165	-0.025	0.005	0.099

Homo sapiens

	A	R	N	D	C	Q	E	G	H	I	L	K	M	F	P	S	T	W	Y	V		∑	∑ of diads
A	9405	5615	4663	5741	1910	4567	7241	6076	2370	6422	9134	6835	2632	4356	5668	8275	6236	928	2945	7310		108329	
R	5424	6512	4513	4819	1693	3945	5658	4583	2231	5308	7566	6422	2183	3780	3823	6367	4466	975	2618	5298		88184	
N	4900	4125	3833	4247	1541	3303	5317	5481	1720	4443	6601	4305	1770	3471	3730	5993	3925	887	2672	5275		77539	
D	6094	4657	3827	7189	1597	3400	8157	6357	1933	5212	7570	5177	2144	4130	4046	6761	4266	1133	3045	6396		93091	
C	1938	1838	1419	1893	735	1342	2062	2161	812	1860	2636	1868	542	1419	1631	2554	1698	347	932	1913		31600	
Q	4144	3740	3520	2788	1484	4727	4083	2854	1604	4085	6784	4907	2149	3101	3231	4699	3595	812	2143	3821		68271	
E	7187	5720	6069	6840	1977	4862	10313	4879	2604	6718	9208	9997	3088	4280	4097	7152	6085	1307	3407	6349		112139	
G	6110	5042	4582	5081	1727	3689	5834	7229	2210	5072	6758	6071	2343	4282	4074	7178	5269	1068	3334	5722		92675	
H	2223	2229	1581	1915	845	1729	2310	2188	1383	2170	3642	1875	849	1891	2025	2716	1936	438	1430	2578		37953	
I	6429	5400	4241	6036	1957	3864	6534	5448	2290	5652	8294	5171	1959	4475	4976	7520	5174	1100	3020	6347		95887	
L	9754	7745	6669	7540	2623	5783	9384	6827	3364	8188	13177	9357	3247	6202	7285	10489	7688	1388	3884	8549		139143	
K	6280	6167	5455	5505	2127	4081	7591	4440	2297	6530	9075	9840	3002	4204	4527	7464	6253	1350	3418	6204		105810	
M	2981	2307	2182	2342	826	1775	2888	2087	901	2526	3627	2953	1355	1945	1986	3727	2583	432	1211	2469		43103	
F	4419	3563	3433	4577	1534	2831	4629	4725	1733	4022	6378	3642	1592	3541	2998	5599	3577	894	2546	4997		71230	
P	5163	3770	3613	4184	1153	3272	5297	5068	1687	4605	6133	4715	1858	3104	5726	6937	5547	680	2112	4823		79447	
S	8499	6513	5914	7231	2352	5049	7912	7685	2682	7298	10214	7354	2960	5234	6307	13515	8597	1222	3613	7621		127772	
T	6359	4218	4125	5139	1985	3122	5713	5243	1964	5780	7657	5124	2093	4003	5272	8340	6589	1049	2706	6675		93156	
W	1068	1021	1060	996	347	741	1008	856	437	1155	1632	1422	584	821	653	1311	1102	238	553	971		17976	
Y	2911	2811	2456	3086	1111	2217	3198	3203	1339	2820	4254	2747	1225	2483	2386	3845	2779	652	1998	3105		50626	
V	7254	5334	4599	6105	2157	4145	7230	5355	2504	6230	9104	6361	2258	4750	5094	7521	5887	1134	3183	7164		103369	
∑	108542	88327	77754	93254	31681	68444	112359	92745	38065	96096	139444	106143	39833	71472	79535	127963	93252	18034	50770	103587		1637300	1637300

	A	R	N	D	C	Q	E	G	H	I	L	K	M	F	P	S	T	W	Y	V
A	0.270	-0.040	-0.098	-0.072	-0.093	0.008	-0.026	-0.010	-0.061	0.010	-0.010	-0.027	-0.001	-0.082	0.074	-0.023	0.011	-0.251	-0.132	0.064
R	-0.075	0.314	0.075	-0.041	-0.008	0.068	-0.067	-0.086	0.085	0.025	0.007	0.116	0.017	-0.018	-0.114	-0.079	-0.117	0.004	-0.044	-0.052
N	-0.048	-0.014	0.040	-0.039	0.027	0.019	-0.001	0.221	-0.047	-0.024	0.000	-0.155	-0.064	0.025	-0.010	-0.011	-0.118	0.038	0.106	0.073
D	-0.013	-0.075	-0.144	0.304	-0.120	-0.135	0.244	0.187	-0.113	-0.047	-0.046	-0.153	-0.055	0.016	-0.111	-0.073	-0.217	0.100	0.053	0.082
C	-0.078	0.075	-0.056	0.050	0.184	0.016	-0.050	0.188	0.100	0.003	-0.021	-0.092	-0.350	0.028	0.061	0.034	-0.058	-0.003	-0.050	-0.044
Q	-0.088	0.015	0.082	-0.333	0.116	0.505	-0.138	-0.304	0.011	0.019	0.154	0.103	0.258	0.040	-0.026	-0.127	-0.078	0.077	0.012	-0.123
E	-0.034	-0.056	0.131	0.069	-0.093	0.036	0.293	-0.264	-0.001	0.021	-0.037	0.319	0.124	-0.134	-0.285	-0.203	-0.048	0.057	-0.020	-0.111
G	-0.006	0.008	0.040	-0.038	-0.038	-0.049	-0.086	0.320	0.025	-0.070	-0.155	0.010	0.038	0.057	-0.100	-0.009	-0.002	0.045	0.149	-0.024
H	-0.124	0.085	-0.131	-0.121	0.140	0.086	-0.120	0.018	0.449	-0.026	0.119	-0.272	-0.084	0.132	0.094	-0.088	-0.110	0.047	0.195	0.071
I	0.011	0.043	-0.071	0.100	0.053	-0.037	-0.007	0.003	0.027	0.004	0.016	-0.184	-0.175	0.067	0.066	0.003	-0.054	0.041	0.016	0.045
L	0.056	0.031	0.009	-0.050	-0.026	-0.006	-0.017	-0.144	0.039	0.003	0.106	0.037	-0.042	0.021	0.075	-0.036	-0.030	-0.099	-0.105	-0.029
K	-0.111	0.077	0.082	-0.091	0.038	-0.081	0.044	-0.300	-0.069	0.050	0.007	0.361	0.154	-0.094	-0.127	-0.102	0.037	0.147	0.041	-0.076
M	0.042	-0.008	0.064	-0.047	-0.010	-0.015	-0.024	-0.157	-0.106	-0.001	-0.012	0.055	0.256	0.033	-0.053	0.101	0.051	-0.094	-0.099	-0.099
F	-0.066	-0.076	0.015	0.121	0.107	-0.050	-0.054	0.158	0.045	-0.039	0.050	-0.237	-0.085	0.130	-0.143	0.006	-0.126	0.131	0.142	0.103
P	-0.020	-0.128	-0.043	-0.078	-0.288	-0.015	-0.029	0.119	-0.091	-0.012	-0.098	-0.088	-0.039	-0.111	0.395	0.111	0.204	-0.252	-0.154	-0.041
S	0.003	-0.057	-0.026	-0.006	-0.050	-0.056	-0.103	0.060	-0.102	-0.027	-0.063	-0.119	-0.049	-0.064	0.016	0.303	0.167	-0.141	-0.092	-0.059
T	0.029	-0.175	-0.070	-0.032	0.096	-0.221	-0.112	-0.006	-0.098	0.056	-0.036	-0.164	-0.080	-0.016	0.153	0.136	0.217	0.022	-0.065	0.124
W	-0.110	0.052	0.216	-0.028	-0.002	-0.014	-0.202	-0.174	0.045	0.091	0.064	0.199	0.289	0.045	-0.291	-0.069	0.074	0.184	-0.008	-0.158
Y	-0.142	0.029	0.021	0.068	0.126	0.046	-0.083	0.111	0.129	-0.052	-0.013	-0.178	-0.005	0.117	-0.030	-0.029	-0.037	0.156	0.241	-0.031
V	0.057	-0.044	-0.065	0.036	0.075	-0.042	0.019	-0.089	0.041	0.027	0.034	-0.052	-0.108	0.051	0.014	-0.072	0.000	-0.004	-0.007	0.091

C. elegans

	A	R	N	D	C	Q	E	G	H	I	L	K	M	F	P	S	T	W	Y	V		∑	∑ of diads
A	17231	6212	6088	6438	2127	6416	8749	9143	3183	6616	12027	7905	3195	4360	7293	11452	8853	1124	3366	8514		140292	
R	6027	7903	5046	5274	1900	5364	6729	5349	2840	5387	9952	6716	2316	3933	4796	8004	5180	1052	3070	5431		102269	
N	6094	4172	5779	4165	1769	3880	5168	6493	2080	4829	7694	4429	2092	3374	4439	7794	4433	978	2857	5441		87960	
D	6768	4691	4275	7277	1740	3753	8087	5967	2108	5581	9322	5351	2297	4275	4608	7286	4642	1129	3423	6290		98870	
C	2117	1964	1579	1954	845	1523	1978	2342	870	1790	3199	1799	619	1364	1647	2793	1643	348	1052	2080		33506	
Q	6524	6205	4042	3823	1454	14432	5833	3976	3588	4358	10068	5120	2448	3077	5152	6502	4660	899	2337	4863		99361	
E	8668	7408	5457	7668	2016	6508	11435	5451	3045	6431	11672	8078	2884	4242	5087	7997	6114	1094	3393	6899		121547	
G	8536	5722	5826	5886	1847	4992	6161	12774	3088	5522	8998	6330	2521	4129	4777	11152	6012	1133	3762	6953		116121	
H	2949	2635	2237	2091	1045	3237	2526	2801	2952	2479	4991	2490	1187	1962	2939	3935	2478	584	1613	2708		49839	
I	6411	5016	4719	5237	2072	4123	5972	5003	2201	5052	8422	5671	2030	4012	4401	7527	5206	984	3328	6038		93425	
L	12818	10348	8002	9554	3083	9753	11510	9429	4730	8183	17499	10408	3732	5874	9342	12867	9099	1672	4809	9745		172457	
K	6840	6991	4671	5799	2037	5402	7588	4791	2667	5690	10215	8502	2541	3708	5753	7865	5724	1111	3410	6276		107581	
M	3616	2712	1935	2618	821	2492	3098	2806	1207	1892	4239	2371	1283	1544	2527	3588	2306	441	1235	2648		45379	
F	4376	3575	3309	4088	1375	2938	4183	4708	1689	3450	6435	3775	1611	2820	2836	5038	3570	808	2392	4644		67620	
P	7927	4561	4466	4627	1373	5095	6508	5964	2774	4364	8360	5502	2082	3114	8917	8231	6356	812	2664	5818		99515	
S	11523	7344	7561	7445	2588	6609	8221	11326	3640	6959	12827	7821	3191	4847	8852	18884	9447	1311	4057	8729		153182	
T	8434	4558	4610	4863	1803	4300	6001	6396	2494	5302	9349	5496	2205	3652	7186	9075	8122	952	2894	6391		104083	
W	1039	1226	891	947	354	964	1024	941	585	986	2034	1067	424	833	744	1522	1028	274	647	964		18494	
Y	3749	3179	2741	3231	1216	2524	3559	3642	1476	2873	5098	2932	1295	2506	2446	3934	2912	676	2105	3366		55460	
V	8852	5965	4936	6076	2091	5222	7389	6935	2725	5801	10326	6133	2398	4151	5850	7986	6421	1149	3194	7618		111218	
∑	140499	102387	88170	99061	33556	99527	121719	116237	49942	93545	172727	107896	42351	67777	99592	153432	104206	18531	55608	111416		1878179	1878179

	A	R	N	D	C	Q	E	G	H	I	L	K	M	F	P	S	T	W	Y	V
A	0.496	-0.208	-0.079	-0.139	-0.164	-0.147	-0.038	0.052	-0.159	-0.055	-0.070	-0.019	0.010	-0.149	-0.020	-0.001	0.129	-0.208	-0.210	0.023
R	-0.238	0.349	0.050	-0.022	0.039	-0.010	0.015	-0.168	0.043	0.056	0.057	0.134	0.004	0.064	-0.123	-0.043	-0.091	0.042	0.014	-0.111
N	-0.077	-0.139	0.336	-0.108	0.118	-0.183	-0.098	0.176	-0.117	0.097	-0.050	-0.132	0.053	0.061	-0.049	0.081	-0.096	0.119	0.093	0.042
D	-0.089	-0.139	-0.082	0.333	-0.015	-0.334	0.233	-0.025	-0.221	0.125	0.025	-0.060	0.030	0.181	-0.129	-0.103	-0.167	0.146	0.156	0.070
C	-0.169	0.073	0.004	0.100	0.345	-0.153	-0.093	0.122	-0.024	0.070	0.037	-0.068	-0.199	0.121	-0.076	0.020	-0.124	0.051	0.059	0.045
Q	-0.130	0.136	-0.143	-0.315	-0.200	1.008	-0.099	-0.436	0.306	-0.127	0.097	-0.109	0.089	-0.153	-0.022	-0.222	-0.168	-0.087	-0.230	-0.192
E	-0.048	0.112	-0.045	0.179	-0.074	0.010	0.373	-0.322	-0.060	0.060	0.043	0.146	0.051	-0.033	-0.237	-0.216	-0.098	-0.092	-0.059	-0.044
G	-0.017	-0.101	0.066	-0.040	-0.116	-0.209	-0.200	0.575	0.000	-0.046	-0.171	-0.052	-0.038	-0.015	-0.254	0.162	-0.069	-0.011	0.090	0.009
H	-0.234	-0.031	-0.045	-0.229	0.160	0.203	-0.246	-0.096	0.801	-0.001	0.085	-0.140	0.055	0.087	0.106	-0.034	-0.110	0.172	0.089	-0.088
I	-0.086	-0.015	0.073	0.061	0.216	-0.183	-0.014	-0.145	-0.121	0.082	-0.020	0.055	-0.037	0.174	-0.118	-0.014	0.004	0.065	0.185	0.086
L	-0.006	0.096	-0.012	0.049	0.001	0.065	0.029	-0.124	0.031	-0.048	0.098	0.049	-0.041	-0.058	0.021	-0.091	-0.050	-0.018	-0.060	-0.049
K	-0.163	0.176	-0.078	0.022	0.058	-0.054	0.085	-0.329	-0.070	0.060	0.032	0.319	0.046	-0.046	0.008	-0.111	-0.042	0.046	0.068	-0.017
M	0.063	0.092	-0.096	0.090	0.013	0.036	0.052	-0.001	0.000	-0.178	0.016	-0.095	0.226	-0.059	0.049	-0.033	-0.088	-0.015	-0.084	-0.016
F	-0.145	-0.031	0.042	0.136	0.129	-0.199	-0.047	0.118	-0.063	0.024	0.034	-0.029	0.055	0.145	-0.235	-0.092	-0.050	0.192	0.178	0.146
P	0.063	-0.173	-0.045	-0.126	-0.258	-0.034	0.009	-0.032	0.047	-0.127	-0.091	-0.038	-0.075	-0.143	0.525	0.012	0.141	-0.190	-0.101	-0.015
S	0.006	-0.128	0.050	-0.082	-0.056	-0.206	-0.189	0.178	-0.112	-0.092	-0.094	-0.118	-0.079	-0.131	0.086	0.411	0.106	-0.142	-0.111	-0.040
T	0.080	-0.219	-0.058	-0.121	-0.031	-0.249	-0.117	-0.007	-0.104	0.023	-0.024	-0.084	-0.062	-0.028	0.264	0.065	0.341	-0.076	-0.063	0.034
W	-0.286	0.196	0.026	-0.030	0.069	-0.016	-0.157	-0.196	0.174	0.068	0.179	0.004	0.017	0.222	-0.276	0.007	0.002	0.407	0.167	-0.129
Y	-0.101	0.050	0.051	0.099	0.205	-0.152	-0.010	0.059	0.001	0.039	0.000	-0.083	0.035	0.225	-0.184	-0.141	-0.055	0.211	0.248	0.023
V	0.062	-0.016	-0.056	0.035	0.051	-0.121	0.025	0.008	-0.082	0.046	0.010	-0.041	-0.045	0.034	-0.008	-0.129	0.040	0.046	-0.030	0.144

Drosophyla melanogaster

	A	R	N	D	C	Q	E	G	H	I	L	K	M	F	P	S	T	W	Y	V		∑	∑ of diads
A	26539	14645	11479	13134	5812	8317	17927	18681	5452	17163	29374	17551	7905	12791	11786	25150	16563	3289	8738	21746		294042	
R	13960	17366	11352	12559	4730	7838	15497	14719	5321	13626	22936	16551	5338	11882	9766	19433	11040	3412	7069	16165		240560	
N	11880	9749	11728	9373	3811	7770	10942	15802	5188	11631	21259	11133	4497	8538	11020	16648	9775	2648	6154	13737		203283	
D	14729	10923	9461	16311	4254	8049	18341	17056	5626	13989	24188	12483	6028	11537	12069	18936	10616	3049	7740	17337		242722	
C	4314	5066	3949	4287	2573	2477	4142	6752	2023	4593	8922	5753	1682	4316	4163	8143	4013	1057	2730	5646		86601	
Q	9787	9332	7513	7358	2616	8702	10464	9336	3660	9058	14558	9456	3967	6247	7111	11748	8067	1910	4149	10165		155204	
E	21531	15265	13737	17644	4011	8884	28714	15634	5213	18526	26401	22091	8565	11496	9526	20619	15811	3356	7891	18202		293117	
G	16616	16277	14403	16560	6040	9545	17221	25191	6324	16955	28272	19665	6766	15983	9995	25670	15001	4057	10701	20430		301672	
H	5385	6315	4300	5010	2355	4376	5576	7652	4343	5386	10022	5140	2290	4505	5218	7579	4262	1208	3468	6670		101060	
I	16677	12531	10555	14513	5121	8725	14665	16763	6320	13393	24468	13972	5020	11111	13455	22936	13373	3476	7728	16985		251787	
L	28962	23952	17787	22958	9122	17342	27809	26942	10706	23213	45252	27871	9485	19168	22316	39042	21992	5242	12175	31264		442600	
K	18339	17108	12414	14483	4791	10002	20883	16433	5753	15895	27578	22577	6772	10280	12960	21279	15262	3818	7507	17393		281527	
M	9545	5830	5252	6708	1649	3764	9203	7257	2209	7000	10060	8313	3655	4576	4431	8845	5498	1219	3291	8283		116588	
F	12212	10160	8609	11942	4418	6764	11190	15034	5118	10278	20613	10959	4365	10434	9496	18934	10537	2579	6394	14268		204304	
P	12223	10400	10835	11523	3303	7539	15072	11891	4353	10071	18212	12339	4195	8757	17207	21167	11581	2817	6060	14044		213589	
S	22348	19812	17526	19951	7912	12704	20664	27577	8324	19977	40337	21691	8455	18939	20148	49274	20030	5464	11357	24937		397427	
T	15670	11338	10025	10720	4544	6899	13817	16615	4534	13440	22595	13475	5492	10651	11169	20743	14998	3145	7648	17049		234567	
W	3310	4069	3515	3095	1099	1826	3469	3313	1088	3644	5555	4071	1422	2631	2057	4595	3014	990	1734	3752		58249	
Y	8754	7026	7011	7630	3083	4361	7791	10510	3154	7282	13042	7979	3417	6541	5623	11076	6827	1976	4864	9070		137017	
V	21943	14122	12450	17379	5658	9630	20199	18881	6629	17219	30072	19062	7178	14454	14477	26581	16789	3728	9981	24509		310941	
∑	294724	241286	203901	243138	86902	155514	293586	302039	101338	252339	443716	282132	106494	204837	213993	398398	235049	58440	137379	311652		4566857	4566857

	A	R	N	D	C	Q	E	G	H	I	L	K	M	F	P	S	T	W	Y	V
A	0.335	-0.059	-0.134	-0.176	0.038	-0.186	-0.053	-0.040	-0.180	0.055	0.028	-0.034	0.142	-0.031	-0.156	-0.020	0.090	-0.135	-0.012	0.080
R	-0.106	0.312	0.055	-0.020	0.033	-0.044	0.002	-0.078	-0.003	0.025	-0.019	0.108	-0.050	0.096	-0.143	-0.077	-0.115	0.103	-0.023	-0.015
N	-0.099	-0.097	0.256	-0.144	-0.015	0.116	-0.178	0.162	0.140	0.035	0.074	-0.120	-0.053	-0.066	0.146	-0.063	-0.068	0.018	0.006	-0.010
D	-0.062	-0.160	-0.136	0.233	-0.082	-0.027	0.162	0.061	0.044	0.042	0.025	-0.183	0.063	0.058	0.059	-0.112	-0.163	-0.019	0.058	0.046
C	-0.259	0.102	0.021	-0.073	0.446	-0.174	-0.296	0.165	0.051	-0.041	0.059	0.073	-0.183	0.105	0.026	0.075	-0.105	-0.047	0.047	-0.046
Q	-0.023	0.129	0.081	-0.116	-0.121	0.499	0.048	-0.095	0.061	0.055	-0.035	-0.014	0.092	-0.108	-0.022	-0.142	0.010	-0.039	-0.118	-0.041
E	0.129	-0.014	0.048	0.123	-0.330	-0.116	0.421	-0.215	-0.221	0.134	-0.076	0.199	0.226	-0.134	-0.366	-0.215	0.047	-0.111	-0.111	-0.094
G	-0.158	0.021	0.067	0.031	0.051	-0.073	-0.119	0.233	-0.057	0.017	-0.036	0.054	-0.039	0.167	-0.347	-0.025	-0.034	0.050	0.165	-0.008
H	-0.192	0.168	-0.048	-0.071	0.203	0.240	-0.153	0.135	0.661	-0.036	0.020	-0.194	-0.029	-0.006	0.097	-0.151	-0.199	-0.068	0.132	-0.033
I	0.026	-0.060	-0.063	0.079	0.067	0.017	-0.099	0.007	0.123	-0.038	0.000	-0.107	-0.157	-0.016	0.131	0.043	0.031	0.076	0.020	-0.012
L	0.014	0.024	-0.105	-0.026	0.080	0.140	-0.023	-0.083	0.086	-0.052	0.051	0.019	-0.084	-0.035	0.073	0.011	-0.035	-0.077	-0.089	0.034
K	0.009	0.140	-0.012	-0.034	-0.112	0.042	0.143	-0.125	-0.082	0.022	0.008	0.261	0.031	-0.206	-0.018	-0.143	0.052	0.058	-0.121	-0.099
M	0.238	-0.055	0.009	0.078	-0.297	-0.053	0.205	-0.061	-0.158	0.083	-0.119	0.143	0.296	-0.133	-0.209	-0.140	-0.087	-0.202	-0.064	0.040
F	-0.077	-0.061	-0.058	0.093	0.128	-0.028	-0.160	0.107	0.121	-0.094	0.038	-0.141	-0.087	0.130	-0.008	0.060	0.002	-0.014	0.040	0.023
P	-0.120	-0.082	0.128	0.013	-0.207	0.036	0.093	-0.172	-0.085	-0.159	-0.131	-0.067	-0.172	-0.090	0.542	0.128	0.052	0.030	-0.059	-0.037
S	-0.138	-0.058	-0.012	-0.059	0.045	-0.063	-0.212	0.048	-0.058	-0.095	0.044	-0.124	-0.092	0.061	0.079	0.352	-0.021	0.072	-0.051	-0.084
T	0.035	-0.089	-0.044	-0.153	0.018	-0.147	-0.087	0.069	-0.138	0.036	-0.009	-0.073	0.004	0.012	0.016	0.014	0.217	0.047	0.081	0.063
W	-0.127	0.279	0.301	-0.002	-0.009	-0.083	-0.076	-0.151	-0.172	0.124	-0.019	0.123	0.046	0.007	-0.283	-0.101	0.005	0.284	-0.010	-0.058
Y	-0.010	-0.030	0.136	0.045	0.168	-0.068	-0.123	0.148	0.037	-0.039	-0.021	-0.059	0.067	0.062	-0.133	-0.076	-0.032	0.120	0.166	-0.030
V	0.089	-0.151	-0.109	0.049	-0.045	-0.095	0.010	-0.085	-0.040	0.002	-0.005	-0.008	-0.010	0.036	-0.006	-0.020	0.048	-0.065	0.065	0.144

Arabidopsis thaliana

	A	R	N	D	C	Q	E	G	H	I	L	K	M	F	P	S	T	W	Y	V		Σ	Σ of diads
A	16600	8881	4633	6985	3318	7373	11333	11652	3544	6852	17496	8508	3290	6631	9248	12123	8123	2063	4022	11527		164202	
R	9349	9812	4796	6311	2821	5728	8891	8704	3404	5835	12970	8477	2481	4605	6871	8763	6231	1624	3621	7575		128869	
N	4753	4228	3375	3423	1954	3543	4933	5299	2054	4734	8341	5020	1909	3535	4976	6161	4230	1147	2760	5151		81526	
D	7125	5616	3743	6159	2381	4203	8071	7638	2512	6116	11634	5995	2478	5173	6678	8366	5448	1580	3680	7243		111839	
C	3031	2981	1746	2244	1504	2314	2776	3766	1391	2145	4990	2515	821	2022	3099	4051	2454	670	1429	3008		48957	
Q	7806	6328	3845	5206	1961	5857	8445	6304	2588	4349	10382	6547	2257	3219	5621	5963	4948	1247	2709	6391		101973	
E	12403	8748	6797	10161	2864	6775	17759	9642	3277	7312	14756	12131	3548	5002	7135	9102	7690	1622	3956	9735		160415	
G	11322	8700	5315	7456	3071	6067	9490	12235	3772	6811	14381	8897	2937	6196	9756	12074	8147	1901	4379	9005		151912	
H	2960	3502	1870	1887	1488	2660	2894	3581	1765	2627	6256	2618	1103	2506	3455	4348	2989	785	1875	3520		54689	
I	6240	5786	4256	4844	2442	5209	6000	5635	2937	5534	10534	5983	2196	4549	5790	8020	5803	1236	3454	6139		102587	
L	16963	14043	7884	10905	5116	12449	15922	14355	5715	9138	25714	13322	4343	8247	12772	16880	11494	2865	6240	13601		227968	
K	9471	7584	5546	7349	2405	5765	11509	7689	3084	6711	12008	11263	3045	4380	6448	8103	6989	1368	4018	8202		132937	
M	5253	2522	2076	2961	934	2035	4191	3283	933	2046	4640	3433	1239	1791	2463	3558	2749	583	1318	3340		51348	
F	5001	4833	3279	4251	2194	4078	4847	5697	2379	4488	9990	4361	1661	3879	4648	7434	4735	1291	2989	5545		87580	
P	10352	7241	4004	6315	2428	5807	10048	11549	2814	4209	11100	6126	2113	4284	11572	10784	6411	1712	3338	8395		130602	
S	11228	9696	5440	7837	3817	7333	10656	12271	3971	6837	16970	8626	3012	6716	10770	16626	8703	2362	4690	10463		168024	
T	8608	5660	3911	5650	2782	4864	7637	8101	2756	5337	11985	5974	2349	4906	7232	8997	6307	1554	3570	8708		116888	
W	1977	1753	1309	1696	639	1138	1871	1786	745	1342	2842	1918	690	1084	1173	1968	1588	474	833	1828		28654	
Y	3619	3982	2641	3326	1594	2862	4116	4246	1743	3338	6602	3651	1359	3119	3209	4950	3532	894	2371	3955		65109	
V	10471	7239	5318	7216	3395	6179	9319	8682	3476	7043	15020	8076	2947	6024	7952	10290	8538	1764	4042	10102		143093	
Σ	164532	129135	81784	112182	49108	102239	160708	152115	54860	102804	228611	133441	45778	87868	130868	168561	117109	28742	65294	143433		2259172	2259172

	A	R	N	D	C	Q	E	G	H	I	L	K	M	F	P	S	T	W	Y	V
A	0.328	-0.055	-0.249	-0.155	-0.073	-0.008	-0.030	0.052	-0.118	-0.087	0.052	-0.131	-0.011	0.038	-0.028	-0.011	-0.047	-0.013	-0.165	0.100
R	-0.004	0.287	0.028	-0.014	0.007	-0.018	-0.031	0.003	0.084	-0.005	-0.005	0.108	-0.051	-0.085	-0.083	-0.093	-0.070	-0.010	-0.028	-0.077
N	-0.222	-0.097	0.134	-0.168	0.098	-0.041	-0.162	-0.035	0.037	0.244	0.011	0.042	0.145	0.109	0.052	0.013	0.001	0.101	0.158	-0.005
D	-0.134	-0.130	-0.079	0.103	-0.021	-0.186	0.014	0.014	-0.078	0.184	0.028	-0.097	0.089	0.173	0.030	0.003	-0.062	0.105	0.130	0.020
C	-0.162	0.063	-0.015	-0.080	0.346	0.043	-0.227	0.133	0.157	-0.038	0.007	-0.140	-0.189	0.060	0.089	0.103	-0.034	0.073	0.010	-0.033
Q	0.050	0.082	0.041	0.028	-0.123	0.238	0.152	-0.085	0.044	-0.065	0.006	0.083	0.088	-0.209	-0.050	-0.244	-0.066	-0.040	-0.084	-0.013
E	0.060	-0.047	0.157	0.243	-0.197	-0.069	0.442	-0.114	-0.173	0.002	-0.095	0.247	0.088	-0.221	-0.264	-0.274	-0.078	-0.230	-0.159	-0.045
G	0.023	0.002	-0.034	-0.012	-0.073	-0.125	-0.130	0.179	0.022	-0.015	-0.067	-0.008	-0.047	0.048	0.103	0.063	0.034	-0.017	-0.003	-0.069
H	-0.297	0.114	-0.057	-0.364	0.225	0.072	-0.296	-0.028	0.284	0.054	0.123	-0.210	-0.005	0.164	0.087	0.064	0.053	0.121	0.171	0.014
I	-0.180	-0.013	0.136	-0.050	0.091	0.115	-0.196	-0.204	0.165	0.170	0.015	-0.013	0.055	0.131	-0.026	0.047	0.087	-0.054	0.153	-0.059
L	0.021	0.075	-0.046	-0.037	0.032	0.188	-0.018	-0.067	0.032	-0.127	0.109	-0.011	-0.062	-0.072	-0.033	-0.008	-0.028	-0.012	-0.054	-0.062
K	-0.022	-0.002	0.142	0.107	-0.184	-0.043	0.196	-0.152	-0.046	0.104	-0.114	0.361	0.123	-0.166	-0.178	-0.202	0.014	-0.212	0.045	-0.029
M	0.340	-0.152	0.110	0.150	-0.178	-0.133	0.137	-0.052	-0.290	-0.133	-0.113	0.124	0.175	-0.109	-0.189	-0.074	0.032	-0.114	-0.119	0.024
F	-0.243	-0.035	0.034	-0.023	0.142	0.028	-0.251	-0.034	0.112	0.119	0.120	-0.171	-0.066	0.130	-0.088	0.129	0.042	0.147	0.166	-0.003
P	0.085	-0.030	-0.166	-0.027	-0.156	-0.018	0.078	0.273	-0.120	-0.345	-0.174	-0.231	-0.225	-0.170	0.425	0.101	-0.054	0.030	-0.123	0.012
S	-0.086	0.010	-0.112	-0.063	0.044	-0.036	-0.115	0.081	-0.027	-0.112	-0.002	-0.140	-0.123	0.027	0.101	0.282	-0.001	0.100	-0.035	-0.019
T	0.011	-0.166	-0.079	-0.027	0.091	-0.084	-0.085	0.029	-0.029	0.003	0.013	-0.145	-0.008	0.076	0.066	0.031	0.040	0.044	0.055	0.160
W	-0.054	0.068	0.233	0.176	0.026	-0.131	-0.086	-0.077	0.068	0.029	-0.020	0.125	0.173	-0.028	-0.347	-0.083	0.067	0.263	0.006	0.005
Y	-0.270	0.068	0.114	0.028	0.119	-0.029	-0.118	-0.032	0.098	0.119	0.002	-0.052	0.030	0.208	-0.162	0.019	0.045	0.076	0.231	-0.044
V	0.005	-0.122	0.026	0.015	0.088	-0.047	-0.088	-0.104	0.000	0.078	0.037	-0.046	0.016	0.079	-0.042	-0.037	0.141	-0.032	-0.023	0.106

Bos taurus

	A	R	N	D	C	Q	E	G	H	I	L	K	M	F	P	S	T	W	Y	V		Σ	Σ of diads
A	7385	3890	2704	4084	1568	3706	5691	5185	1966	3416	8522	4252	1907	3142	3892	6545	3869	807	2010	6137		80678	
R	4298	5382	2675	3613	1480	3312	4841	3925	1912	2958	6732	4705	1583	2507	3318	5350	3424	792	2039	4151		68997	
N	2733	2611	2076	1991	1086	2111	2620	3341	1251	2628	4458	2800	1210	1790	2748	3901	2763	549	1392	2765		46824	
D	3702	3225	2280	4117	1410	2302	4974	4260	1571	3461	6260	3384	1441	2745	3425	5464	3283	843	1939	4213		64299	
C	1587	1643	967	1296	765	1240	1466	1786	744	1170	2650	1500	534	1089	1499	2306	1420	328	787	1906		26683	
Q	3867	3720	2384	2952	1187	4175	4431	3012	1742	2511	5568	3494	1439	1833	2802	4324	3315	686	1620	3322		58384	
E	5501	5314	3512	5844	1578	3930	9471	4652	1938	3617	7922	6065	2179	2685	3204	5622	4209	866	2079	5091		85279	
G	4821	4089	2860	3832	1469	3273	4557	5561	2057	3343	6665	4472	1720	3136	3548	6561	3906	957	2327	4816		73970	
H	1762	1989	1251	1235	858	1581	1610	1975	1396	1727	3380	1696	821	1338	1954	2944	1966	480	1118	1856		32937	
I	3338	3128	2355	2703	1320	2695	3205	2977	1585	2933	5479	3156	1304	2248	3145	4906	3462	675	1726	3148		55488	
L	7774	6790	4679	5852	2626	6859	8312	6515	3405	5017	12835	7475	2540	4424	6246	10085	6339	1385	3412	6707		119277	
K	4748	4634	2815	4204	1234	3416	5913	3854	1868	3392	6415	5903	1793	2066	3610	4820	3959	747	1992	4236		71619	
M	2636	1480	1320	1826	591	1435	2604	1861	709	1225	2722	1964	969	1083	1321	2473	1636	363	822	1918		30958	
F	2552	2374	1850	2235	1157	2082	2492	2767	1307	2530	4925	2330	1069	2033	2348	4265	2692	645	1528	2787		45968	
P	4732	3207	2361	3576	1217	3027	4558	4405	1843	2352	5822	3077	1310	2189	5454	6111	3342	685	1829	4495		65592	
S	7027	5566	3452	5285	2360	4623	6203	6547	2586	4167	9741	5156	2137	3724	6676	12347	5613	1144	2681	6624		103659	
T	4828	3038	2288	3466	1578	2954	4541	4389	1855	2881	6519	3159	1276	2597	4200	5463	3651	757	1691	4792		65923	
W	869	926	640	843	314	639	889	725	345	666	1392	928	404	668	570	1167	835	211	429	864		14324	
Y	1806	2103	1464	1736	908	1594	2047	2189	954	1857	3519	1863	869	1541	1745	3034	1997	415	1300	2061		35002	
V	4844	4031	3005	3804	2041	3545	4984	4119	1994	3737	8034	4444	1810	3254	3974	6274	4393	1039	2374	5188		76888	
Σ	80810	69140	46938	64494	26747	58499	85409	74045	33028	55588	119560	71823	28315	46092	65679	103962	66074	14374	35095	77077		1222749	1222749

	A	R	N	D	C	Q	E	G	H	I	L	K	M	F	P	S	T	W	Y	V
A	0.326	-0.159	-0.136	-0.041	-0.118	-0.041	0.010	0.059	-0.103	-0.071	0.077	-0.108	0.021	0.033	-0.107	-0.047	-0.119	-0.161	-0.142	0.188
R	-0.059	0.322	0.010	-0.007	-0.020	0.003	0.004	-0.063	0.026	-0.059	-0.002	0.149	-0.009	-0.037	-0.111	-0.092	-0.085	-0.024	0.029	-0.047
N	-0.124	-0.014	0.144	-0.215	0.059	-0.059	-0.222	0.164	-0.011	0.211	-0.027	0.018	0.110	0.014	0.089	-0.020	0.088	-0.003	0.035	-0.065
D	-0.138	-0.120	-0.079	0.194	0.002	-0.290	0.102	0.090	-0.100	0.169	-0.004	-0.110	-0.033	0.124	-0.008	-0.001	-0.057	0.109	0.049	0.039
C	-0.105	0.085	-0.058	-0.082	0.271	-0.029	-0.240	0.100	0.032	-0.036	0.016	-0.044	-0.146	0.079	0.045	0.016	-0.015	0.045	0.027	0.125
Q	0.002	0.119	0.062	-0.042	-0.073	0.402	0.083	-0.160	0.099	-0.055	-0.025	0.019	0.062	-0.183	-0.113	-0.138	0.049	0.000	-0.034	-0.102
E	-0.024	0.097	0.070	0.262	-0.167	-0.037	0.464	-0.104	-0.173	-0.069	-0.051	0.191	0.098	-0.180	-0.357	-0.254	-0.091	-0.146	-0.163	-0.054
G	-0.014	-0.023	0.007	-0.018	-0.097	-0.078	-0.126	0.216	0.029	-0.006	-0.082	0.029	0.004	0.118	-0.113	0.042	-0.023	0.096	0.092	0.032
H	-0.211	0.066	-0.011	-0.341	0.175	0.003	-0.357	-0.010	0.451	0.143	0.048	-0.132	0.074	0.075	0.099	0.050	0.099	0.215	0.168	-0.112
I	-0.094	-0.003	0.100	-0.080	0.084	0.015	-0.190	-0.121	0.056	0.151	0.010	-0.032	0.015	0.072	0.054	0.039	0.144	0.034	0.080	-0.105
L	-0.014	0.007	0.022	-0.072	0.006	0.184	-0.002	-0.103	0.055	-0.078	0.096	0.065	-0.084	-0.016	-0.025	-0.006	-0.017	-0.012	-0.003	-0.114
K	0.003	0.135	0.024	0.107	-0.239	-0.003	0.167	-0.118	-0.035	0.041	-0.088	0.339	0.078	-0.268	-0.064	-0.234	0.023	-0.120	-0.031	-0.064
M	0.253	-0.168	0.105	0.112	-0.136	-0.032	0.186	-0.007	-0.165	-0.139	-0.106	0.077	0.301	-0.075	-0.230	-0.062	-0.022	-0.003	-0.078	-0.017
F	-0.174	-0.091	0.047	-0.081	0.140	-0.055	-0.253	-0.006	0.051	0.191	0.091	-0.147	0.004	0.160	-0.050	0.087	0.080	0.177	0.147	-0.039
P	0.088	-0.145	-0.064	0.033	-0.165	-0.036	-0.005	0.103	0.039	-0.237	-0.097	-0.225	-0.148	-0.122	0.437	0.091	-0.059	-0.118	-0.029	0.084
S	0.025	-0.052	-0.142	-0.034	0.040	-0.070	-0.155	0.042	-0.079	-0.123	-0.040	-0.166	-0.116	-0.048	0.181	0.337	0.002	-0.063	-0.104	0.014
T	0.103	-0.205	-0.101	-0.003	0.090	-0.065	-0.014	0.095	0.041	-0.039	0.011	-0.204	-0.179	0.044	0.171	-0.026	0.025	-0.023	-0.112	0.143
W	-0.086	0.134	0.152	0.110	0.002	-0.070	-0.118	-0.179	-0.115	0.022	-0.006	0.098	0.197	0.213	-0.300	-0.043	0.076	0.226	0.043	-0.044
Y	-0.248	0.061	0.086	-0.062	0.171	-0.049	-0.178	0.032	0.009	0.154	0.028	-0.099	0.070	0.155	-0.075	0.019	0.054	0.009	0.258	-0.068
V	-0.048	-0.076	0.018	-0.064	0.194	-0.037	-0.075	-0.123	-0.041	0.067	0.066	-0.016	0.016	0.116	-0.038	-0.041	0.056	0.139	0.073	0.068

Danio rerio

	A	R	N	D	C	Q	E	G	H	I	L	K	M	F	P	S	T	W	Y	V		Σ	Σ of diads
A	8197	3647	2475	3510	1451	3028	5633	5119	1418	3289	7011	4373	1606	2758	3910	5461	3729	777	1862	5416		74670	
R	3799	4354	2305	2826	1269	2660	3859	3656	1449	2783	5129	3876	1235	1994	2825	3881	2817	763	1791	3206		56477	
N	2531	2084	1870	1831	1010	1713	2665	3024	935	2407	3918	2600	1014	1658	2474	3401	2164	547	1374	2704		41924	
D	3335	2429	2136	3206	1246	1944	4247	3826	1116	3069	5194	3054	1238	2382	2936	4201	2660	660	1852	3510		54241	
C	1308	1345	951	1156	645	973	1378	1573	578	1102	2065	1422	405	911	1327	1927	1226	306	680	1328		22606	
Q	3385	2684	2011	2247	1030	3187	3659	2605	1300	2196	4542	3113	1082	1588	2652	2950	2380	583	1378	2778		47350	
E	5478	4291	3389	4920	1359	3357	8834	4270	1577	3595	6761	5979	1806	2413	3106	4551	3732	810	2057	4681		76966	
G	4651	3706	2690	3515	1360	2640	4601	5168	1595	3199	5700	4384	1480	2657	3966	5589	3744	794	2129	3898		67466	
H	1442	1470	1015	970	672	1120	1379	1545	915	1277	2654	1349	536	1022	1585	2027	1292	350	852	1441		24913	
I	3226	2732	2281	2579	1150	2518	2999	2673	1322	2684	4861	3136	1157	2117	2849	4069	3127	575	1572	2973		50600	
L	6836	5485	3841	4857	2097	5507	7144	5412	2583	4237	10267	6274	2032	3379	5537	7524	5228	1079	2820	5432		97571	
K	4749	3872	2800	3730	1208	2870	5913	3796	1512	3379	5840	5871	1629	1995	3294	4086	3522	701	2020	3942		66729	
M	2270	1176	1067	1466	414	1021	2142	1477	487	1069	2114	1941	623	852	1136	1710	1342	281	694	1608		24890	
F	2219	1987	1527	1991	918	1878	2264	2494	1033	2111	4006	2074	864	1629	1953	3325	2328	470	1379	2468		38918	
P	4694	2889	2027	2876	1098	2472	4302	5201	1372	2123	4818	3073	1058	1864	5380	5005	3062	616	1628	3748		59306	
S	5525	4128	2944	4170	1800	3461	5150	5466	1909	3617	7197	4452	1535	2985	5288	8845	4595	1009	2229	5017		81322	
T	4364	2523	2043	2871	1334	2217	3808	3739	1215	2823	5206	3164	1154	2198	3379	4546	3390	763	1764	4375		56876	
W	749	724	585	695	248	546	801	643	302	674	1264	911	368	510	468	908	767	188	364	712		12427	
Y	1643	1853	1418	1652	772	1406	2014	2057	822	1699	2990	1820	711	1438	1545	2534	1795	397	1149	1825		31540	
V	4370	3195	2650	3284	1587	2926	4305	3775	1535	3365	6258	4041	1368	2650	3771	4986	4065	782	2013	4459		65385	
Σ	74771	56574	42025	54352	22668	47444	77097	67519	24975	50698	97795	66907	22901	39000	59381	81526	56965	12451	31607	65521		1052177	1052177

	A	R	N	D	C	Q	E	G	H	I	L	K	M	F	P	S	T	W	Y	V
A	0.435	-0.096	-0.186	-0.094	-0.103	-0.106	0.029	0.066	-0.223	-0.090	0.010	-0.082	-0.012	-0.004	-0.075	-0.058	-0.081	-0.129	-0.186	0.153
R	-0.055	0.360	0.022	-0.032	0.042	0.044	-0.070	0.009	0.078	0.022	-0.023	0.076	0.005	-0.049	-0.121	-0.120	-0.082	0.132	0.054	-0.093
N	-0.163	-0.079	0.110	-0.168	0.112	-0.099	-0.142	0.117	-0.062	0.175	0.005	-0.025	0.105	0.065	0.045	0.046	-0.048	0.098	0.087	0.035
D	-0.145	-0.183	-0.014	0.135	0.064	-0.230	0.066	0.095	-0.143	0.161	0.030	-0.122	0.047	0.170	-0.042	0.000	-0.099	0.028	0.128	0.038
C	-0.206	0.101	0.052	-0.010	0.281	-0.047	-0.184	0.081	0.074	0.012	-0.017	-0.011	-0.195	0.084	0.039	0.095	0.002	0.134	0.001	-0.058
Q	0.006	0.053	0.061	-0.085	0.010	0.401	0.053	-0.154	0.146	-0.038	0.032	0.033	0.049	-0.100	-0.008	-0.218	-0.074	0.040	-0.032	-0.060
E	0.002	0.036	0.098	0.213	-0.199	-0.033	0.449	-0.146	-0.147	-0.031	-0.056	0.200	0.075	-0.167	-0.335	-0.270	-0.110	-0.117	-0.117	-0.024
G	-0.030	0.021	-0.002	0.009	-0.066	-0.142	-0.072	0.177	-0.004	-0.016	-0.095	0.022	0.008	0.061	0.041	0.067	0.025	-0.005	0.049	-0.075
H	-0.205	0.093	0.020	-0.283	0.225	-0.003	-0.280	-0.034	0.437	0.062	0.136	-0.161	-0.012	0.101	0.120	0.049	-0.043	0.172	0.130	-0.074
I	-0.109	0.004	0.121	-0.013	0.053	0.099	-0.212	-0.195	0.096	0.096	0.033	-0.026	0.049	0.121	-0.002	0.037	0.132	-0.041	0.034	-0.058
L	-0.014	0.045	-0.014	-0.037	-0.002	0.225	-0.001	-0.146	0.109	-0.104	0.124	0.011	-0.044	-0.068	0.006	-0.005	-0.010	-0.068	-0.039	-0.112
K	0.001	0.076	0.049	0.079	-0.174	-0.047	0.190	-0.120	-0.046	0.050	-0.060	0.325	0.115	-0.215	-0.134	-0.235	-0.025	-0.119	0.008	-0.053
M	0.250	-0.129	0.071	0.131	-0.259	-0.095	0.161	-0.078	-0.193	-0.115	-0.090	0.204	0.140	-0.080	-0.212	-0.120	-0.004	-0.047	-0.075	0.037
F	-0.220	-0.052	-0.018	-0.010	0.091	0.068	-0.231	-0.001	0.112	0.118	0.102	-0.177	0.020	0.122	-0.117	0.098	0.100	0.020	0.165	0.018
P	0.108	-0.099	-0.156	-0.063	-0.152	-0.079	-0.010	0.312	-0.026	-0.297	-0.135	-0.205	-0.199	-0.165	0.475	0.085	-0.047	-0.130	-0.090	0.015
S	-0.045	-0.058	-0.098	-0.007	0.027	-0.058	-0.146	0.046	-0.011	-0.080	-0.049	-0.150	-0.142	-0.010	0.142	0.339	0.043	0.047	-0.092	-0.009
T	0.077	-0.192	-0.106	-0.023	0.085	-0.146	-0.090	0.024	-0.105	0.030	-0.015	-0.134	-0.070	0.042	0.051	0.031	0.096	0.125	0.032	0.211
W	-0.165	0.080	0.164	0.079	-0.077	-0.026	-0.128	-0.215	0.024	0.118	0.090	0.142	0.308	0.102	-0.405	-0.059	0.131	0.246	-0.025	-0.083
Y	-0.311	0.089	0.118	0.014	0.128	-0.011	-0.138	0.016	0.093	0.112	0.020	-0.097	0.035	0.207	-0.142	0.036	0.050	0.062	0.193	-0.073
V	-0.061	-0.096	0.015	-0.028	0.119	-0.008	-0.107	-0.106	-0.011	0.066	0.029	-0.028	-0.040	0.089	0.022	-0.016	0.138	0.011	0.025	0.091

Gallus gallus

	A	R	N	D	C	Q	E	G	H	I	L	K	M	F	P	S	T	W	Y	V		∑	∑ of diads
A	8435	4350	3359	4499	1760	4186	6723	5475	2178	4401	9297	5609	2291	3645	4627	7756	4896	904	2344	6358		93093	
R	4396	5992	3374	3986	1535	3802	5473	4436	2185	3881	7392	6021	1791	2933	3912	6200	4152	815	2360	4094		78730	
N	3409	3216	2898	2818	1383	2746	3990	4104	1647	3683	6046	4049	1576	2377	3590	5519	3396	715	2089	3746		62997	
D	4154	3706	3135	4981	1718	2884	6087	4853	1812	4526	7507	4553	1850	3407	4172	6610	3860	1002	2619	4753		78189	
C	1696	1716	1378	1602	959	1471	1882	2311	885	1557	3039	1987	648	1423	1804	2846	1885	373	924	1789		32175	
Q	4464	4173	3255	3566	1403	4995	5580	3672	1965	3462	6705	4635	1745	2325	3435	5106	3727	703	2087	3831		70834	
E	6583	5972	5051	7024	2125	4901	11890	5642	2346	5104	9291	8424	2561	3280	3864	7127	5343	1048	2756	5900		106232	
G	5438	4599	3966	4621	1823	3743	5711	6625	2401	4524	7284	6169	2090	3789	3741	7903	4895	1023	2865	5144		88354	
H	1910	2276	1665	1428	905	2114	2126	2249	1625	2087	4155	2202	971	1565	2462	3525	2332	554	1302	2151		39604	
I	4296	3923	3114	3618	1739	3650	4444	3849	2226	3941	7070	4717	1709	2769	4076	6179	4214	846	2223	4121		72724	
L	8780	7304	6078	7059	3014	8022	9635	7526	4093	6134	14321	9354	2919	5092	7374	11923	7298	1495	4007	7828		139256	
K	6038	6000	4081	5375	1901	4633	8141	4942	2379	4719	8442	8420	2396	2759	5061	6806	4967	934	2765	5573		96332	
M	3106	1698	1723	2290	712	1768	3131	2077	901	1499	3030	2629	968	1329	1765	3085	1912	373	1083	2170		37249	
F	3094	2824	2337	2896	1374	2587	2894	3400	1558	2954	5831	3056	1189	2342	2854	5166	3272	640	1857	3306		55431	
P	5356	3784	3150	4118	1584	3564	5793	5367	2116	3183	6671	4263	1658	2918	6648	7623	4345	722	2420	5396		80679	
S	7845	6152	5055	6714	2839	5655	7817	8035	3365	5606	11807	6962	2602	4502	8410	14657	6879	1330	3451	7454		127137	
T	5481	3530	3074	4192	1875	3277	5687	5488	2095	3781	7329	4302	1756	3069	5155	7110	4362	939	2267	5565		80334	
W	978	963	802	968	369	689	1047	869	391	834	1602	1211	462	681	591	1230	888	271	502	867		16215	
Y	2166	2403	1990	2148	1063	2089	2661	2835	1204	2501	4212	2544	1066	1930	2300	3901	2512	508	1596	2476		44105	
V	5617	4341	3655	4486	2169	4200	5691	4686	2365	4487	8552	5479	1914	3433	4939	7205	5306	1064	2710	5521		87820	
∑	93242	78922	63140	78389	32250	70976	106403	88441	39737	72864	139583	96586	34162	55568	80780	127477	80441	16259	44227	88043		1487490	1487490

	A	R	N	D	C	Q	E	G	H	I	L	K	M	F	P	S	T	W	Y	V
A	0.368	-0.127	-0.162	-0.087	-0.137	-0.059	0.010	-0.011	-0.133	-0.036	0.062	-0.075	0.069	0.047	-0.089	-0.028	-0.028	-0.118	-0.166	0.143
R	-0.116	0.361	0.010	-0.040	-0.106	0.012	-0.029	-0.054	0.038	0.006	0.001	0.164	-0.010	-0.003	-0.089	-0.085	-0.025	-0.054	0.008	-0.129
N	-0.147	-0.039	0.080	-0.164	0.012	-0.090	-0.122	0.091	-0.022	0.177	0.022	-0.010	0.086	0.010	0.048	0.022	-0.003	0.038	0.109	0.005
D	-0.165	-0.113	-0.057	0.190	0.013	-0.257	0.085	0.043	-0.142	0.167	0.023	-0.109	0.030	0.154	-0.018	-0.014	-0.091	0.159	0.119	0.027
C	-0.173	0.005	0.009	-0.057	0.318	-0.043	-0.201	0.189	0.029	-0.012	0.007	-0.050	-0.131	0.169	0.032	0.032	0.080	0.059	-0.035	-0.063
Q	0.005	0.105	0.079	-0.046	-0.090	0.391	0.096	-0.137	0.038	-0.002	0.009	0.008	0.070	-0.129	-0.113	-0.173	-0.027	-0.097	-0.009	-0.090
E	-0.011	0.058	0.113	0.227	-0.081	-0.034	0.448	-0.113	-0.190	-0.019	-0.070	0.200	0.049	-0.191	-0.401	-0.245	-0.073	-0.103	-0.136	-0.064
G	-0.018	-0.019	0.056	-0.008	-0.050	-0.119	-0.101	0.232	0.017	0.044	-0.129	0.073	0.030	0.138	-0.249	0.043	0.024	0.058	0.087	-0.017
H	-0.262	0.080	-0.010	-0.379	0.053	0.112	-0.287	-0.046	0.429	0.073	0.112	-0.155	0.065	0.056	0.135	0.038	0.085	0.247	0.100	-0.086
I	-0.059	0.017	0.009	-0.058	0.098	0.051	-0.158	-0.116	0.136	0.101	0.035	-0.001	0.023	0.019	0.032	-0.009	0.069	0.062	0.028	-0.044
L	0.006	-0.012	0.028	-0.039	-0.002	0.188	-0.033	-0.095	0.096	-0.106	0.092	0.034	-0.091	-0.021	-0.025	-0.001	-0.031	-0.018	-0.033	-0.052
K	0.000	0.160	-0.002	0.057	-0.094	0.008	0.167	-0.148	-0.079	0.000	-0.068	0.297	0.080	-0.266	-0.033	-0.193	-0.048	-0.120	-0.035	-0.023
M	0.285	-0.152	0.086	0.154	-0.126	-0.005	0.161	-0.064	-0.099	-0.197	-0.143	0.083	0.124	-0.046	-0.136	-0.034	-0.052	-0.088	-0.022	-0.016
F	-0.116	-0.041	-0.007	-0.009	0.134	-0.022	-0.315	0.031	0.051	0.084	0.114	-0.164	-0.068	0.123	-0.053	0.084	0.088	0.055	0.119	0.008
P	0.057	-0.123	-0.084	-0.032	-0.099	-0.077	0.004	0.112	-0.018	-0.216	-0.127	-0.206	-0.111	-0.032	0.417	0.098	-0.004	-0.200	0.009	0.122
S	-0.016	-0.092	-0.065	0.002	0.030	-0.070	-0.151	0.061	-0.009	-0.105	-0.010	-0.170	-0.115	-0.054	0.197	0.297	0.001	-0.044	-0.091	-0.009
T	0.085	-0.189	-0.104	-0.010	0.074	-0.157	-0.010	0.139	-0.024	-0.040	-0.028	-0.193	-0.049	0.022	0.167	0.032	0.004	0.067	-0.052	0.157
W	-0.039	0.113	0.153	0.125	0.048	-0.116	-0.102	-0.104	-0.102	0.049	0.052	0.140	0.216	0.117	-0.399	-0.122	0.013	0.425	0.040	-0.102
Y	-0.244	0.027	0.061	-0.079	0.106	-0.007	-0.170	0.078	0.022	0.146	0.018	-0.118	0.051	0.158	-0.041	0.032	0.052	0.052	0.196	-0.053
V	0.020	-0.071	-0.020	-0.031	0.130	0.002	-0.099	-0.108	0.008	0.042	0.037	-0.040	-0.052	0.045	0.035	-0.044	0.111	0.103	0.037	0.060

Xenopus laevis

	A	R	N	D	C	Q	E	G	H	I	L	K	M	F	P	S	T	W	Y	V		Σ	Σ of diads
A	11751	5893	3797	6448	1308	5307	7970	8597	2136	6516	12857	5232	2829	3777	3839	5581	5025	1119	2582	7906		110470	
R	5691	4351	2275	3837	733	3884	4676	4401	2023	4208	7347	2754	1613	3015	2687	3289	2796	914	2322	4888		67704	
N	3787	2414	1649	2167	449	1842	2305	3167	1040	2789	4096	1728	1047	1531	2532	2122	2050	534	1164	2907		41320	
D	5778	3464	2356	3310	705	2222	4213	4516	1362	4680	6145	2921	1574	2454	2528	3294	3030	984	2250	4857		62643	
C	1170	805	402	709	188	640	713	1447	460	729	1136	428	169	519	638	779	525	209	411	794		12871	
Q	5843	3885	1470	2278	461	4816	2830	3809	1630	2715	6729	2054	1299	1984	2819	2533	2274	834	1374	4265		55902	
E	6571	4880	2843	3109	619	4730	4338	4605	1834	4211	8138	3871	1923	2434	2365	3360	3419	818	1663	5030		70761	
G	7157	4877	2857	4522	1252	3526	5544	6868	2215	6000	8673	5015	2559	3726	2660	4818	4307	1212	2714	6902		87404	
H	2308	1540	1018	1284	471	1625	1268	2240	957	1733	2851	1016	556	1246	1793	1560	1346	475	894	1824		28005	
I	7212	4038	2854	4508	720	2416	4521	5647	1555	3819	6115	3039	1357	2200	3184	4157	4070	647	1842	4513		68414	
L	13357	7286	4798	7080	1379	5373	6752	9135	2764	6473	13111	5358	2675	4659	6524	7681	7110	1429	3067	8486		124497	
K	5032	3331	1949	2471	322	2750	3068	3468	1116	2738	5663	2504	1169	1554	2596	2633	2863	468	1232	3739		50666	
M	3472	1858	1196	1421	292	1342	1548	2266	688	1696	3347	1494	965	902	1446	2162	1862	253	625	2515		31350	
F	3751	2142	1987	2829	739	1357	2340	3763	960	2818	3498	1615	995	1712	1665	3089	2608	554	1387	2783		42592	
P	4779	2305	1644	2991	433	2704	4268	3657	1249	2843	5881	1877	1200	1843	1824	2613	2609	656	1502	4312		51190	
S	5835	3873	2015	3459	651	3012	3769	5866	1741	3353	7089	2321	1356	2332	2962	3453	3206	961	1790	4482		63526	
T	5482	3223	1766	3190	662	2492	3461	4873	1417	3395	7829	2092	1196	2170	3521	2978	3103	625	1408	4266		59149	
W	1088	913	449	679	169	1318	663	723	484	648	2278	455	313	611	601	640	440	228	419	1064		14183	
Y	2690	2225	1018	1743	497	1906	1370	2513	1034	1562	3480	1050	586	1267	1558	1886	1561	537	817	1939		31239	
V	8064	4666	3087	4764	871	2868	5486	6115	1543	5605	8544	4267	2335	2814	3549	5110	4966	786	1866	6802		84108	
Σ	110818	67969	41430	62799	12921	56130	71103	87676	28208	68531	124807	51091	27716	42750	51291	63738	59170	14243	31329	84274		1157994	1157994

	A	R	N	D	C	Q	E	G	H	I	L	K	M	F	P	S	T	W	Y	V
A	0.106	-0.096	-0.040	0.074	0.059	-0.009	0.161	0.027	-0.231	-0.003	0.077	0.071	0.068	-0.077	-0.243	-0.086	-0.116	-0.194	-0.146	-0.017
R	-0.130	0.091	-0.063	0.044	-0.030	0.168	0.118	-0.153	0.204	0.049	0.007	-0.081	-0.005	0.188	-0.110	-0.125	-0.213	0.093	0.237	-0.008
N	-0.043	-0.005	0.109	-0.033	-0.026	-0.084	-0.096	0.012	0.033	0.131	-0.084	-0.054	0.057	0.004	0.325	-0.069	-0.029	0.049	0.040	-0.034
D	-0.037	-0.060	0.050	-0.026	0.009	-0.312	0.091	-0.049	-0.114	0.233	-0.094	0.055	0.049	0.059	-0.093	-0.046	-0.055	0.245	0.283	0.063
C	-0.051	0.064	-0.136	0.016	0.269	0.026	-0.103	0.395	0.383	-0.044	-0.200	-0.283	-0.600	0.088	0.113	0.095	-0.225	0.278	0.166	-0.165
Q	0.088	0.169	-0.308	-0.286	-0.302	0.575	-0.193	-0.105	0.180	-0.198	0.111	-0.183	-0.030	-0.039	0.130	-0.195	-0.228	0.193	-0.096	0.047
E	-0.030	0.161	0.116	-0.211	-0.243	0.321	-0.002	-0.151	0.062	0.006	0.065	0.215	0.127	-0.071	-0.282	-0.148	-0.056	-0.062	-0.141	-0.024
G	-0.156	-0.051	-0.090	-0.047	0.250	-0.184	0.032	0.037	0.040	0.148	-0.083	0.263	0.202	0.144	-0.375	0.001	-0.036	0.120	0.138	0.082
H	-0.149	-0.065	0.016	-0.168	0.410	0.180	-0.305	0.055	0.339	0.045	-0.057	-0.196	-0.187	0.187	0.368	0.012	-0.061	0.321	0.165	-0.111
I	0.097	0.006	0.154	0.195	-0.058	-0.317	0.073	0.086	-0.069	-0.058	-0.187	0.007	-0.188	-0.138	0.049	0.099	0.152	-0.263	-0.005	-0.098
L	0.114	-0.003	0.074	0.047	-0.007	-0.116	-0.124	-0.031	-0.093	-0.129	-0.023	-0.025	-0.108	0.014	0.168	0.114	0.111	-0.069	-0.094	-0.065
K	0.037	0.113	0.073	-0.106	-0.563	0.113	-0.014	-0.101	-0.101	-0.091	0.036	0.113	-0.037	-0.185	0.146	-0.057	0.101	-0.286	-0.107	0.014
M	0.146	0.010	0.064	-0.179	-0.181	-0.124	-0.218	-0.046	-0.104	-0.090	-0.009	0.077	0.252	-0.249	0.041	0.225	0.150	-0.421	-0.305	0.097
F	-0.083	-0.155	0.265	0.203	0.441	-0.420	-0.111	0.154	-0.078	0.112	-0.272	-0.151	-0.024	0.085	-0.125	0.276	0.181	0.056	0.185	-0.108
P	-0.025	-0.265	-0.108	0.075	-0.277	0.086	0.306	-0.058	0.002	-0.064	0.064	-0.185	-0.021	-0.025	-0.218	-0.075	-0.003	0.041	0.081	0.146
S	-0.041	0.038	-0.120	0.004	-0.085	-0.022	-0.034	0.199	0.118	-0.114	0.035	-0.189	-0.114	-0.006	0.051	-0.013	-0.012	0.207	0.041	-0.031
T	-0.032	-0.074	-0.181	-0.006	0.003	-0.140	-0.048	0.084	-0.017	-0.031	0.205	-0.221	-0.169	-0.006	0.296	-0.089	0.026	-0.152	-0.128	-0.009
W	-0.221	0.092	-0.122	-0.125	0.066	0.651	-0.273	-0.396	0.337	-0.259	0.399	-0.319	-0.081	0.154	-0.044	-0.199	-0.499	0.268	0.088	0.030
Y	-0.106	0.193	-0.093	0.028	0.355	0.230	-0.337	0.061	0.307	-0.169	0.033	-0.272	-0.244	0.094	0.119	0.092	-0.022	0.335	-0.034	-0.159
V	0.002	-0.056	0.026	0.043	-0.075	-0.352	0.060	-0.041	-0.284	0.119	-0.059	0.140	0.148	-0.098	-0.049	0.099	0.145	-0.275	-0.198	0.105

Yersinia pestis

	A	R	N	D	C	Q	E	G	H	I	L	K	M	F	P	S	T	W	Y	V		Σ	Σ of diads
A	13544	8186	10622	8866	2484	7071	10132	9381	3889	12381	17747	13022	3910	7680	8048	17385	12189	1690	5812	10776		184815	
R	7637	9637	9372	8295	2052	5904	9185	7313	3520	9785	14397	12533	2809	6590	5666	12678	8285	1597	5319	7587		150161	
N	10758	7504	16263	13612	2526	6569	14335	11650	4153	13818	17610	14524	3752	8895	8194	19923	11983	2256	6819	11679		206823	
D	10368	7070	10764	15299	2298	5811	16589	9702	3760	14630	18769	11804	3711	9132	8195	16730	10120	2128	6937	11546		195363	
C	2281	1893	2085	2295	1087	1686	2206	2801	1168	3202	4673	2652	832	2414	1937	3988	2353	546	1624	2557		44280	
Q	6618	6737	7972	7104	1641	9781	9236	5811	2970	8277	13628	9143	2600	5922	5574	9864	6709	1391	4996	6857		132831	
E	11485	10011	14910	14334	2239	8605	19752	9102	4044	14368	20992	17990	4196	9108	6934	16335	12287	2105	7427	11270		217494	
G	9289	7053	9929	9085	2532	5793	9517	10473	3820	11552	15225	12591	3459	7582	5560	15921	10225	2044	6081	9935		167666	
H	3594	3466	4116	4267	1226	2825	4261	3873	2452	4818	7462	4484	1256	3334	3808	6664	3975	869	2783	3987		73520	
I	12159	9508	13291	13379	3244	8003	13559	10556	4871	14864	21541	15474	4170	10390	11299	20103	12885	2573	7287	12357		221513	
L	17768	15084	19598	17880	4529	14652	20192	15107	7386	19735	32091	25185	5779	14431	16085	28215	17783	3002	10488	17172		322162	
K	12366	13537	15325	14043	2889	9265	17950	10127	5054	16302	23638	23040	4389	11059	9996	19200	13615	2622	9183	13326		246926	
M	4608	2998	4526	4410	847	2565	4531	3918	1317	4497	6171	5380	1644	2915	2688	6371	4485	665	2021	4200		70757	
F	7981	5766	8890	9019	2091	6705	9186	8422	3664	10501	14885	10318	2722	7747	6558	12568	8640	1751	5555	8571		151540	
P	7619	6036	8342	7321	1396	6649	9666	6438	3467	9850	14254	9729	2799	6632	8579	14762	9431	1494	4814	8855		148133	
S	16296	13366	19660	15715	3619	12009	15729	14884	6580	19217	28606	22115	5801	13222	13304	36850	21302	2769	8676	15821		305541	
T	11919	8088	12327	10217	2318	6606	11196	10380	4040	12825	18655	14100	3367	8864	10291	19575	15192	2137	5929	12070		200096	
W	1774	1705	2267	2175	787	1307	2169	1766	724	2143	3331	3006	643	1833	1045	3011	1673	520	1325	2043		35247	
Y	5767	4985	6584	7128	1908	4628	6860	6334	2872	7300	11281	7560	2242	5754	4770	9123	6123	1346	4904	6411		113880	
V	11348	7917	10483	11285	2695	6723	11703	9813	3993	11954	17979	13144	3458	8410	9770	16794	11127	1882	6167	11813		188458	
Σ	185179	150547	207326	195729	44408	133157	217954	167851	73744	222019	322935	247794	63539	151914	148301	306060	200382	35387	114147	188833		3377206	3377206

	A	R	N	D	C	Q	E	G	H	I	L	K	M	F	P	S	T	W	Y	V
A	0.290	-0.006	-0.066	-0.189	0.022	-0.030	-0.163	0.021	-0.037	0.019	0.004	-0.041	0.117	-0.079	-0.008	0.037	0.106	-0.136	-0.072	0.042
R	-0.075	0.364	0.017	-0.048	0.038	-0.003	-0.054	-0.020	0.071	-0.009	0.003	0.129	-0.006	-0.025	-0.152	-0.071	-0.073	0.015	0.047	-0.101
N	-0.053	-0.206	0.248	0.127	-0.074	-0.216	0.071	0.125	-0.084	0.016	-0.116	-0.044	-0.036	-0.045	-0.103	0.061	-0.024	0.040	-0.025	0.010
D	-0.033	-0.208	-0.108	0.301	-0.111	-0.282	0.274	-0.001	-0.126	0.130	0.005	-0.194	0.010	0.038	-0.046	-0.057	-0.136	0.039	0.049	0.055
C	-0.062	-0.042	-0.265	-0.112	0.624	-0.035	-0.259	0.241	0.189	0.095	0.099	-0.203	-0.001	0.192	-0.004	-0.006	-0.110	0.163	0.082	0.032
Q	-0.096	0.129	-0.023	-0.080	-0.062	0.625	0.075	-0.128	0.024	-0.054	0.070	-0.064	0.040	-0.009	-0.045	-0.199	-0.161	-0.001	0.107	-0.080
E	-0.038	0.032	0.110	0.129	-0.245	0.003	0.342	-0.172	-0.161	0.005	0.009	0.120	0.025	-0.072	-0.320	-0.188	-0.049	-0.079	0.010	-0.076
G	0.010	-0.058	-0.036	-0.067	0.138	-0.132	-0.128	0.229	0.042	0.047	-0.052	0.023	0.092	0.005	-0.281	0.047	0.027	0.151	0.071	0.058
H	-0.115	0.056	-0.092	0.001	0.238	-0.026	-0.108	0.058	0.424	-0.003	0.060	-0.185	-0.096	0.008	0.165	0.000	-0.093	0.120	0.113	-0.031
I	0.001	-0.038	-0.023	0.041	0.108	-0.087	-0.053	-0.042	0.007	0.021	0.017	-0.049	0.001	0.042	0.150	0.001	-0.020	0.103	-0.027	-0.002
L	0.006	0.049	-0.009	-0.043	0.067	0.143	-0.029	-0.058	0.049	-0.071	0.041	0.063	-0.048	-0.004	0.128	-0.034	-0.072	-0.117	-0.038	-0.048
K	-0.091	0.207	0.011	-0.019	-0.117	-0.050	0.119	-0.192	-0.065	0.004	0.001	0.240	-0.057	-0.004	-0.081	-0.153	-0.073	0.013	0.096	-0.035
M	0.172	-0.051	0.041	0.073	-0.094	-0.084	-0.008	0.108	-0.160	-0.034	-0.092	0.036	0.211	-0.088	-0.145	-0.006	0.066	-0.109	-0.168	0.060
F	-0.040	-0.158	-0.045	0.027	0.048	0.115	-0.063	0.112	0.102	0.053	0.027	-0.075	-0.046	0.128	-0.015	-0.089	-0.040	0.098	0.081	0.011
P	-0.064	-0.090	-0.086	-0.159	-0.333	0.130	0.011	-0.134	0.069	0.011	0.006	-0.111	0.004	-0.005	0.277	0.095	0.070	-0.038	-0.039	0.067
S	-0.028	-0.019	0.047	-0.119	-0.105	-0.003	-0.226	-0.020	-0.014	-0.044	-0.021	-0.014	0.009	-0.039	-0.008	0.286	0.161	-0.145	-0.174	-0.077
T	0.083	-0.098	0.004	-0.127	-0.127	-0.178	-0.143	0.043	-0.078	-0.025	-0.025	-0.040	-0.112	-0.015	0.158	0.076	0.247	0.019	-0.132	0.076
W	-0.086	0.082	0.047	0.063	0.529	-0.061	-0.048	0.008	-0.061	-0.078	-0.012	0.150	-0.031	0.145	-0.393	-0.059	-0.223	0.342	0.106	0.036
Y	-0.080	-0.018	-0.060	0.077	0.242	0.030	-0.069	0.113	0.144	-0.025	0.035	-0.100	0.045	0.116	-0.047	-0.123	-0.099	0.120	0.242	0.007
V	0.094	-0.059	-0.099	0.033	0.084	-0.100	-0.039	0.047	-0.030	-0.036	-0.002	-0.051	-0.025	-0.008	0.166	-0.017	-0.005	-0.048	-0.032	0.114

Saccharomyces cerevisiae

	A	R	N	D	C	Q	E	G	H	I	L	K	M	F	P	S	T	W	Y	V		∑	∑ of diads
A	21683	7196	3658	6381	2169	3476	7176	10156	2508	5479	10857	5388	3199	4613	6585	9712	6773	1386	2976	9895		131266	
R	6760	7551	2800	4193	1477	2852	4994	5800	2159	3908	7257	4671	1748	3293	4032	5933	3441	1202	2416	5235		81722	
N	3702	2467	2171	2342	855	1702	2483	4234	1159	2551	4265	2631	1206	2114	3033	3789	2408	766	1573	3422		48873	
D	6430	3723	2353	5517	1018	2264	5519	6909	1827	3781	6466	3420	1816	3045	3885	4692	3148	914	2306	5276		74309	
C	1739	1498	845	1196	644	778	1202	2321	757	1142	2126	1305	498	968	1228	2011	1120	300	747	1411		23836	
Q	3798	3002	1859	2108	831	3517	3107	3186	1267	2475	4863	2548	1168	1800	2789	3181	2026	612	1395	3343		48875	
E	7548	4795	3074	4867	1110	3190	7848	5148	1776	4201	8301	5138	2218	3104	3192	4654	3445	1049	2503	5796		82957	
G	9196	6464	3805	6164	1855	3441	5859	14570	2516	5002	8810	5426	2445	4431	4034	8348	5225	1762	3240	7363		109956	
H	2610	2163	1176	1575	554	1330	1545	2944	1593	1494	3092	1308	626	1416	2157	2092	1402	473	995	2085		32630	
I	5193	3399	2735	3504	1362	2317	3459	4606	1635	3321	6473	3286	1459	2949	3587	5237	3633	840	2013	4864		65872	
L	11479	7825	4119	6418	2206	5256	7108	8419	3132	5454	13662	6631	2619	5196	7733	9581	5927	1509	3408	8929		126611	
K	5828	4402	2823	3917	964	2808	5189	4448	1505	3694	6213	5401	1641	2533	3426	4528	3171	889	2199	4848		70427	
M	4256	1765	1282	2143	562	1256	2663	2441	822	1538	2940	1806	949	1375	1681	2451	1550	376	972	2428		35256	
F	4521	3095	1921	3230	1177	2043	2863	4423	1446	2695	5675	2510	1295	2826	2932	4599	2694	860	1924	4293		57022	
P	7289	4431	2617	3681	1080	2399	4544	5255	1543	3015	6146	3205	1457	2995	7674	6707	3860	1028	1970	4886		75782	
S	8670	5934	3801	5162	2096	3266	5089	8413	2127	4973	9282	5077	2424	4586	6146	12522	5686	1455	2808	6528		106045	
T	6623	3274	2436	3191	1151	1836	3557	5315	1271	3582	6072	3240	1553	2977	3806	5534	4441	772	1916	5242		67789	
W	1379	1363	744	987	341	580	969	1176	490	941	1652	962	476	888	873	1353	955	332	593	1193		18247	
Y	2899	2412	1641	2203	858	1419	2163	3318	1053	2079	3648	1985	1016	1876	1918	2750	1918	538	1496	2842		40032	
V	9950	5145	3194	5666	1649	3277	5800	7017	2149	4705	9108	4697	2196	4206	5186	6744	5063	1226	2734	8517		98229	
∑	131553	81904	49054	74445	23959	49007	83137	110099	32735	66030	126908	70635	32009	57191	75897	106418	67886	18289	40184	98396		1395736	1395736

	A	R	N	D	C	Q	E	G	H	I	L	K	M	F	P	S	T	W	Y	V
A	0.561	-0.068	-0.232	-0.093	-0.038	-0.282	-0.086	-0.019	-0.205	-0.125	-0.095	-0.209	0.061	-0.154	-0.081	-0.030	0.059	-0.216	-0.239	0.067
R	-0.131	0.454	-0.025	-0.039	0.052	-0.006	0.026	-0.106	0.119	0.011	-0.024	0.122	-0.070	-0.017	-0.097	-0.049	-0.144	0.116	0.026	-0.096
N	-0.219	-0.151	0.234	-0.107	0.019	-0.008	-0.159	0.094	0.011	0.098	-0.041	0.062	0.073	0.054	0.132	0.017	0.013	0.179	0.111	-0.007
D	-0.085	-0.158	-0.104	0.331	-0.226	-0.142	0.221	0.164	0.047	0.073	-0.044	-0.095	0.064	0.000	-0.039	-0.189	-0.138	-0.063	0.075	0.007
C	-0.256	0.069	0.009	-0.061	0.454	-0.073	-0.167	0.211	0.303	0.013	-0.019	0.079	-0.093	-0.009	-0.054	0.101	-0.035	-0.040	0.085	-0.175
Q	-0.193	0.046	0.079	-0.212	-0.010	0.718	0.065	-0.191	0.100	0.068	0.090	0.030	0.041	-0.107	0.048	-0.158	-0.160	-0.045	-0.009	-0.030
E	-0.035	-0.015	0.053	0.095	-0.249	0.091	0.463	-0.240	-0.091	0.068	0.096	0.202	0.153	-0.091	-0.346	-0.307	-0.158	-0.036	0.047	-0.009
G	-0.120	0.002	-0.016	0.050	-0.017	-0.115	-0.111	0.519	-0.025	-0.039	-0.126	-0.025	-0.031	-0.017	-0.394	-0.004	-0.023	0.201	0.023	-0.051
H	-0.164	0.122	0.025	-0.100	-0.011	0.149	-0.230	0.134	0.733	-0.033	0.041	-0.233	-0.178	0.057	0.195	-0.173	-0.124	0.101	0.057	-0.098
I	-0.179	-0.129	0.167	-0.003	0.186	0.002	-0.126	-0.121	0.057	0.064	0.078	-0.014	-0.035	0.089	0.001	0.042	0.126	-0.027	0.060	0.046
L	-0.039	0.052	-0.077	-0.051	0.015	0.167	-0.059	-0.171	0.053	-0.094	0.171	0.034	-0.103	0.002	0.116	-0.008	-0.038	-0.095	-0.067	0.000
K	-0.130	0.063	0.131	0.042	-0.226	0.127	0.213	-0.222	-0.093	0.103	-0.030	0.416	0.016	-0.130	-0.111	-0.170	-0.077	-0.037	0.081	-0.024
M	0.247	-0.159	0.034	0.131	-0.074	0.015	0.238	-0.130	-0.006	-0.081	-0.087	0.012	0.160	-0.049	-0.131	-0.092	-0.101	-0.206	-0.043	-0.023
F	-0.173	-0.078	-0.042	0.060	0.184	0.020	-0.171	-0.017	0.078	-0.001	0.090	-0.140	-0.010	0.190	-0.056	0.056	-0.029	0.141	0.159	0.066
P	0.020	-0.004	-0.018	-0.094	-0.186	-0.104	0.007	-0.129	-0.141	-0.173	-0.114	-0.179	-0.176	-0.036	0.622	0.149	0.046	0.035	-0.102	-0.089
S	-0.142	-0.048	0.020	-0.091	0.141	-0.131	-0.216	0.006	-0.156	-0.009	-0.038	-0.055	-0.003	0.054	0.064	0.437	0.097	0.046	-0.084	-0.136
T	0.036	-0.195	0.022	-0.125	-0.011	-0.260	-0.127	-0.006	-0.224	0.111	-0.015	-0.057	-0.001	0.069	0.032	0.068	0.298	-0.140	-0.018	0.092
W	-0.221	0.241	0.149	0.014	0.085	-0.100	-0.115	-0.202	0.135	0.086	-0.004	0.041	0.129	0.172	-0.128	-0.028	0.073	0.328	0.121	-0.075
Y	-0.264	0.026	0.154	0.031	0.222	0.009	-0.097	0.049	0.115	0.093	0.002	-0.020	0.101	0.134	-0.127	-0.104	-0.015	0.025	0.261	0.007
V	0.072	-0.114	-0.078	0.078	-0.022	-0.051	-0.009	-0.099	-0.070	0.012	0.020	-0.057	-0.026	0.044	-0.030	-0.105	0.058	-0.049	-0.034	0.207

Oriza sativa
