# Supplementary material for: Positive Evolutionary Selection of an HD Motif on Alzheimer Precursor Protein Orthologues Suggests a Functional Role
Source: PLoS Comput Biol. 2012 Feb 2;8(2):e1002356. doi: 10.1371/journal.pcbi.1002356 (PMC3271017; doi:10.1371/journal.pcbi.1002356)
Supplement: Table S2 — The p values of the different amino acid dyads in the neutral evolution simulation. The values of Panels A, B, and C were calculated from different regions (1–70, 10–54 and 1–96, respectively) of the alignment shown in Figure 1. Table A, shows the p values of 10 000 simulated runs. Table B, contains the sum of amino acid dyads in the examined regions of the 41 CAEDs. The first amino acids of the dyads are represented on the vertical axis while the second amino acids are represented on the horizontal axis. (PDF) [file pcbi.1002356.s003.pdf]

Panel A

	A	R	N	D	C	Q	E	G	H	I	L	K	M	F	P	S	T	W	Y	V
A	471	2415	2272	3172	10000	4705	184	9801	2209	9049	8586	5438	7827	10000	6169	7808	7874	10000	6394	3442
R	3112	2164	2756	6875	10000	8367	2722	2697	407	9158	10000	7051	6623	10000	2472	5203	10000	10000	10000	7185
N	8380	8692	10000	9013	10000	10000	2033	8935	7014	1130	9464	897	6121	7493	10000	6507	1475	10000	10000	8099
D	617	2388	7479	3282	10000	6763	4632	10000	7631	4245	4782	6468	7102	6517	10000	749	6940	10000	7306	688
C	10000	10000	10000	10000	10000	10000	10000	10000	10000	10000	10000	10000	10000	10000	10000	10000	10000	10000	10000	10000
Q	9119	1996	8013	8705	10000	2826	2122	8727	1174	10000	9208	1021	5545	7010	2797	4439	2129	10000	10000	2271
E	4178	5368	1897	315	10000	4704	599	3945	4917	1375	8945	8809	7575	303	41	2953	8467	10000	5767	1102
G	10000	3538	8964	9295	10000	10000	6879	10000	2259	10000	3220	9476	10000	4169	8131	1879	10000	10000	1625	10000
H	2573	1664	5324	0	10000	18	3911	3928	675	7595	10000	10000	10000	1619	10000	3445	10000	10000	10000	2878
I	9100	8281	8907	7455	10000	5472	7894	8538	7935	7674	9645	1133	10000	8422	10000	1003	10000	10000	3723	8460
L	9894	4045	10000	9226	10000	6673	10000	5516	8663	8148	10000	9811	10000	10000	2690	7894	2071	10000	8197	2130
K	10000	10000	4039	8590	10000	10000	4156	2158	6353	8618	1634	8437	65	10000	5910	9615	938	10000	10000	4567
M	7748	10000	6147	424	10000	2073	7551	7228	2728	6611	7579	10000	10000	10000	5355	7196	1079	10000	4093	10000
F	334	1880	10000	6412	10000	4937	4695	4643	6166	8246	10000	8594	1065	589	10000	2645	6076	10000	3783	10000
P	1146	7891	5499	6198	10000	1507	4619	2257	5838	5435	8869	3216	5362	10000	6189	3734	2067	10000	10000	1828
S	5860	6082	1475	7706	10000	8808	389	2020	2784	9543	4172	7728	4725	2340	3476	2443	5092	10000	2657	5077
T	4616	1633	1246	9287	10000	10000	1583	9321	1988	7972	9619	3815	6523	3728	6011	8254	1293	10000	10000	2846
W	10000	10000	10000	10000	10000	10000	10000	10000	10000	10000	10000	10000	10000	10000	10000	10000	10000	10000	10000	10000
Y	6416	2963	10000	10000	10000	10000	1233	5455	2324	7039	10000	10000	10000	10000	10000	2698	10000	10000	10000	10000
V	4249	4000	10000	338	10000	2023	320	2442	648	10000	4927	1923	7167	929	10000	6345	9482	10000	5849	10000

A,

	A	R	N	D	C	Q	E	G	H	I	L	K	M	F	P	S	T	W	Y	V
A	32	18	17	17	0	5	34	1	17	2	4	8	1	0	3	4	3	0	2	16
R	14	15	11	3	0	1	17	17	23	1	0	3	1	0	18	5	0	0	0	3
N	2	1	0	1	0	0	17	1	1	19	1	21	1	1	0	3	16	0	0	2
D	30	16	2	16	0	2	10	0	1	10	12	4	1	2	0	25	3	0	1	27
C	0	0	0	0	0	0	0	0	0	0	0	0	0	0	0	0	0	0	0	0
Q	1	12	1	1	0	4	14	1	15	0	1	19	1	1	5	4	10	0	0	12
E	14	5	18	32	0	4	27	13	3	22	3	2	1	26	36	15	2	0	2	25
G	0	12	1	1	0	0	4	0	15	0	20	1	0	5	1	21	0	0	16	0
H	12	15	2	41	0	31	5	4	20	1	0	0	0	10	0	5	0	0	0	6
I	2	2	1	3	0	3	3	2	1	2	1	21	0	1	0	21	0	0	3	2
L	1	12	0	2	0	3	0	9	1	3	0	1	0	0	14	4	20	0	1	20
K	0	0	6	2	0	0	11	20	2	2	22	2	25	0	3	1	21	0	0	9
M	1	0	1	20	0	3	1	1	2	1	1	0	0	0	1	1	11	0	1	0
F	28	14	0	2	0	2	4	4	1	1	0	1	9	21	0	10	2	0	2	0
P	22	1	2	2	0	12	4	19	1	3	1	7	1	0	1	6	14	0	0	17
S	7	4	19	3	0	1	29	21	8	1	12	3	2	11	6	15	5	0	5	7
T	8	18	19	1	0	0	21	1	11	2	1	9	1	4	2	2	18	0	0	13
W	0	0	0	0	0	0	0	0	0	0	0	0	0	0	0	0	0	0	0	0
Y	2	4	0	0	0	0	17	2	3	1	0	0	0	0	0	5	0	0	0	0
V	13	10	0	31	0	15	31	18	21	0	10	20	1	20	0	5	1	0	2	0

B,

Panel B

	A	R	N	D	C	Q	E	G	H	I	L	K	M	F	P	S	T	W	Y	V
A	136	1582	10000	2352	10000	6062	164	9678	560	7832	10000	9411	6345	10000	7947	7236	10000	10000	4874	1668
R	2188	10000	10000	10000	10000	10000	2357	2148	296	10000	10000	6566	5361	10000	1627	10000	10000	10000	10000	5568
N	8718	10000	10000	10000	10000	10000	10000	10000	4872	577	10000	5682	4833	10000	10000	7978	10000	10000	10000	6696
D	318	1730	10000	1958	10000	5046	2915	10000	5773	3293	3420	4942	10000	7258	10000	602	6890	10000	6122	2879
C	10000	10000	10000	10000	10000	10000	10000	10000	10000	10000	10000	10000	10000	10000	10000	10000	10000	10000	10000	10000
Q	10000	1014	5964	10000	10000	1837	1160	10000	332	10000	10000	10000	10000	5560	2591	7194	6796	10000	10000	1412
E	3107	4408	10000	1777	10000	3452	224	6587	3917	1046	7818	8783	10000	894	381	2371	6931	10000	4273	588
G	10000	8809	8177	8839	10000	10000	5456	10000	908	10000	2825	10000	10000	4464	10000	1312	10000	10000	1351	10000
H	4109	771	5043	0	10000	484	2942	2356	825	5587	10000	10000	10000	799	10000	1905	10000	10000	10000	2053
I	8015	8885	10000	7970	10000	7368	6241	7611	7014	6002	9008	577	10000	10000	10000	457	10000	10000	10000	8570
L	9657	8308	10000	9633	10000	10000	10000	4507	7581	9026	10000	10000	10000	10000	6215	6668	1100	10000	10000	9308
K	10000	10000	4417	6995	10000	10000	7324	9369	4360	8923	8439	6728	23	10000	5064	8955	357	10000	10000	9105
M	6381	10000	10000	159	10000	1327	10000	10000	1782	5122	10000	10000	10000	10000	4062	10000	822	10000	10000	10000
F	10000	1491	10000	5335	10000	10000	3410	3949	10000	6424	10000	10000	589	4458	10000	1616	6931	10000	2139	10000
P	623	10000	6166	6912	10000	1136	5043	1921	4208	3487	7904	2247	3952	10000	4936	2662	4717	10000	10000	7626
S	7278	10000	10000	7617	10000	7426	94	1677	1813	10000	3272	7452	5768	1443	10000	1836	8654	10000	1859	3767
T	5322	1078	530	8788	10000	10000	742	9166	1082	8329	9107	10000	10000	3078	4736	8801	620	10000	10000	8646
W	10000	10000	10000	10000	10000	10000	10000	10000	10000	10000	10000	10000	10000	10000	10000	10000	10000	10000	10000	10000
Y	6816	2229	10000	10000	10000	10000	581	10000	10000	5458	10000	10000	10000	10000	10000	1846	10000	10000	10000	10000
V	2607	4628	10000	119	10000	892	2473	10000	283	10000	8131	893	5679	10000	10000	5683	9015	10000	10000	10000

A,

	A	R	N	D	C	Q	E	G	H	I	L	K	M	F	P	S	T	W	Y	V
A	31	17	0	16	0	2	30	1	17	2	0	1	1	0	1	3	0	0	2	16
R	14	0	0	0	0	0	11	17	20	0	0	2	1	0	18	0	0	0	0	3
N	1	0	0	0	0	0	0	0	1	19	0	2	1	0	0	1	0	0	0	2
D	29	16	0	13	0	2	10	0	1	8	12	4	0	1	0	21	2	0	1	12
C	0	0	0	0	0	0	0	0	0	0	0	0	0	0	0	0	0	0	0	0
Q	0	12	1	0	0	3	12	0	14	0	0	0	0	1	3	1	1	0	0	11
E	12	4	0	14	0	3	25	3	2	19	3	1	0	17	20	11	2	0	2	22
G	0	1	1	1	0	0	4	0	15	0	20	0	0	4	0	19	0	0	16	0
H	2	12	1	41	0	12	3	4	4	1	0	0	0	9	0	4	0	0	0	4
I	2	1	0	2	0	1	3	2	1	2	1	21	0	0	0	21	0	0	0	1
L	1	2	0	1	0	0	0	9	1	1	0	0	0	0	2	4	20	0	0	1
K	0	0	3	2	0	0	2	1	2	1	2	2	23	0	3	1	21	0	0	1
M	1	0	0	20	0	3	0	0	2	1	0	0	0	0	1	0	10	0	0	0
F	0	14	0	2	0	0	4	3	0	1	0	0	9	1	0	10	1	0	2	0
P	21	0	1	1	0	9	2	19	1	3	1	6	1	0	1	6	2	0	0	1
S	3	0	0	2	0	1	29	19	5	0	11	2	1	11	0	13	1	0	5	6
T	4	17	19	1	0	0	19	1	11	1	1	0	0	3	2	1	18	0	0	1
W	0	0	0	0	0	0	0	0	0	0	0	0	0	0	0	0	0	0	0	0
Y	1	4	0	0	0	0	17	0	0	1	0	0	0	0	0	5	0	0	0	0
V	13	5	0	31	0	14	13	0	20	0	2	20	1	0	0	4	1	0	0	0

B,

Panel C

	A	R	N	D	C	Q	E	G	H	I	L	K	M	F	P	S	T	W	Y	V
A	569	2514	2805	3768	10000	5820	371	5431	2065	2824	6270	5132	4960	5149	7138	8924	1554	10000	7734	4721
R	4018	2408	3050	7302	10000	8623	2605	3110	416	9611	10000	7616	7472	10000	1999	5921	10000	10000	10000	8435
N	7447	8998	10000	9176	10000	10000	2099	9378	7345	1837	9344	1148	7123	8391	10000	7152	1879	10000	10000	3173
D	875	2570	7899	3205	10000	7151	4757	10000	7724	5141	5818	7030	7775	7390	10000	867	7568	10000	7742	1017
C	10000	10000	10000	10000	10000	10000	10000	6643	10000	4885	10000	10000	10000	10000	10000	10000	10000	10000	10000	7428
Q	9522	2257	8312	8896	10000	3169	2133	9217	943	10000	9662	1091	6445	7903	2666	5229	2576	10000	10000	3137
E	4860	5839	1930	445	10000	5147	733	4757	5076	1945	9485	9105	8107	452	67	3327	8908	10000	6281	1459
G	3868	4368	9414	9611	10000	10000	7780	3262	2359	4588	4473	9784	10000	5469	8670	2614	10000	10000	2040	425
H	2870	1582	5529	0	10000	38	3971	4598	472	8403	9040	10000	10000	1810	10000	3747	10000	10000	10000	3695
I	43	8290	9516	8429	3751	6955	8809	2395	8654	1794	9995	2083	8783	8791	10000	2100	2372	10000	4865	2358
L	9000	5523	10000	9667	10000	8035	10000	7283	9150	9244	9992	3550	23	10000	3515	5245	3008	10000	9319	4456
K	10000	10000	4667	8812	10000	10000	4493	2748	6658	9422	2699	8805	122	10000	6392	9747	1050	10000	10000	5951
M	1617	7599	6996	522	10000	2756	8244	6775	3311	7053	1142	10000	10000	3432	6195	8353	1567	10000	5548	731
F	2	2172	10000	7350	10000	5964	5627	6339	6774	5928	10000	6809	1445	899	10000	3616	1794	10000	5194	3477
P	1380	8083	6054	6684	10000	1663	4920	2358	6037	6623	9439	3621	6154	10000	6420	4151	2174	10000	4010	2348
S	7135	6873	1707	8128	6433	9126	526	2649	2955	9109	5947	8491	6300	3331	4049	3014	5222	10000	3459	6248
T	5472	1888	748	9536	10000	10000	1639	9748	2153	7917	572	4582	5754	5388	6717	8126	1758	10000	10000	230
W	10000	10000	10000	10000	10000	10000	10000	10000	10000	10000	10000	10000	10000	10000	10000	10000	10000	10000	10000	10000
Y	7720	3610	10000	10000	10000	10000	1237	6872	2510	8666	10000	10000	10000	1258	10000	3615	10000	10000	10000	7464
V	4739	5171	10000	554	10000	2459	545	625	921	2465	7017	2479	821	10	10000	8180	9876	10000	2210	3497

A,

	A	R	N	D	C	Q	E	G	H	I	L	K	M	F	P	S	T	W	Y	V
A	35	20	17	17	0	5	34	16	17	23	17	13	4	8	3	4	24	0	2	19
R	14	15	11	3	0	1	17	17	23	1	0	3	1	0	18	5	0	0	0	3
N	4	1	0	1	0	0	17	1	1	19	2	21	1	1	0	3	16	0	0	15
D	30	16	2	16	0	2	10	0	1	10	12	4	1	2	0	25	3	0	1	27
C	0	0	0	0	0	0	0	1	0	2	0	0	0	0	0	0	0	0	0	1
Q	1	12	1	1	0	4	14	1	15	0	1	19	1	1	7	4	10	0	0	12
E	14	5	18	32	0	4	27	13	3	22	3	2	1	26	36	15	2	0	2	25
G	21	12	1	1	0	0	4	21	15	17	23	1	0	6	1	21	0	0	16	36
H	12	15	2	41	0	31	5	4	20	1	1	0	0	10	0	5	0	0	0	6
I	38	3	1	3	3	3	3	24	1	24	1	21	1	2	0	21	19	0	4	24
L	7	12	0	2	0	3	0	11	1	5	1	20	34	0	14	15	21	0	1	22
K	0	0	6	2	0	0	11	20	2	2	22	2	25	0	3	1	22	0	0	9
M	15	1	1	20	0	3	1	2	2	2	20	0	0	3	1	1	11	0	1	20
F	40	15	0	2	0	2	4	4	1	5	0	3	10	21	0	10	17	0	2	14
P	22	1	2	2	0	12	4	19	1	3	1	7	1	0	1	6	14	0	2	17
S	7	4	19	3	1	1	29	21	8	3	13	3	2	11	6	15	7	0	5	9
T	10	18	22	1	0	0	21	1	11	4	32	9	2	4	2	3	18	0	0	33
W	0	0	0	0	0	0	0	0	0	0	0	0	0	0	0	0	0	0	0	0
Y	2	4	0	0	0	0	17	2	3	1	0	0	0	15	0	5	0	0	0	2
V	19	10	0	31	0	15	31	35	21	24	14	21	20	38	0	5	1	0	14	21

B,
