# Supplementary material for: Positive Evolutionary Selection of an HD Motif on Alzheimer Precursor Protein Orthologues Suggests a Functional Role
Source: PLoS Comput Biol. 2012 Feb 2;8(2):e1002356. doi: 10.1371/journal.pcbi.1002356 (PMC3271017; doi:10.1371/journal.pcbi.1002356)
Supplement: Table S3 — Metal-binding prediction results on the CAEDs of APPOs. (PDF) [file pcbi.1002356.s004.pdf]

Species	Accession number	Region	SVMProt	Metallopred
Acyrthosiphon pisum	XP_001947569.1	499-556	I,Z,Me	Me,TM,Co
Culex quinquefasciatus	XP_001864483.1	517-575	N,Me	Me,TM,Co
Brugia malayi	XP_001899252	621-677	M,Z	Me,TM,Z
Caenorhabditis briggsae	XP_002644641.1	545-615	Z	Me,AM,C
Loligo pealei	ABI84193.2	484-544	M,C, Me	Me,TM,Z
Aplysia californica	AAT07668.3	544-594	Z,C,Me	Me,AM,C
Aedes aegypti	EAT42567.1	624-682	N,Z	Me,TM,Co
Drosophila simulans 1	EDX16764.1	351-411	N,M,Z,Me	Me,TM,Cb
Drosophila yakuba 2	EDX00795.1	704-764	N,M,Z,Me	Me,TM,Cb
Anopheles gambiae	XP_312126.4	745-803	N,Z,Me	Me,TM,Co
Nematostella vectensis	EDO45291.1	451-507	C,Z,Me	Me,TM,Z
Drosophila willistoni 3	XP_002067462.1	775-834	C,Z,M,	Me,TM,Co
Drosophila persimilis 4	XP_002027785.1	844-903	Z,M,	Me,TM,Co
Drosophila pseudoobscura 5	XP_001354498.2	807-866	Z,M,	Me,TM,Co
Drosophila virilis 6	XP_002055698.1	743-802	C,Z,M,	Me,TM,Co
Drosophila grimshawi 7	XP_001992447.1	780-839	Z,M,	Me,TM,Co
Drosophila erecta 8	XP_001982404.1	758-818	N,M,Z,Me	Me,TM,Cb
Drosophila ananassae 9	XP_001966309.1	767-827	N,M,Z,Ma	Me,TM,Co
Nasonia vitripennis	XP_001601635.1	645-700	Z,C,Me	Me,TM,Z
Culex quinquefasciatus	XP_001864483.1	517-575	N,Z,Me	Me,TM,Co
Pediculus humanus corporis	XP_002426948.1	108-162	Z	Me,TM,Co
Manduca sexta	AAY25024.2	601-660	Co	Me,TM,Z
Rattus norvegicus	NP_062161.1	631-700	I,C,Me	Me,AM,C
Mus musculus	Q53ZT3	631-700	I,C,Me	Me,AM,C
Monodelphis domestica	XP_001373948.1	689-758	C,M, Me	Me,AM,C
Equus caballus	XP_001499900.2	612-681	I,C,Me	Me,AM,C
Sus scrofa,	ABB82034.1	612-681	I,C,Me	Me,AM,C
Gallus gallus,	AAG00594.1	612-681	I,C,Me	Me,AM,C
Canis lupus familiaris	AAX81908.1	631-700	I,C,Me	Me,AM,C
Macaca fascicularis	BAD51938.1	612-681	I,C,Me	Me,AM,C
Ailuropoda melanoleuca	XP_002920108.1	630-699	I,C,Me	Me,AM,C
Oryctolagus cuniculus	XP_002716819.1	630-699	I,C,Me	Me,AM,C
Pan troglodytes	AAV74286.1	631-700	I,C,Me	Me,AM,C
Callithrix jacchus	XP_002761374.1	631-700	I,C,Me	Me,TM,Co
Stenella coeruleoalba	AAX81912.1	610-679	I,Z,Me	Me,AM,C
Xenopus (Silurana) tropicalis	AAH75266.1	611-680	I,C,Me	Me,TM,Cb
Xenopus laevis,	AAH70668.1	610-679	Z,C,Me	Me,TM,Co
Pongo abelii	NP_001127014.1	556-625	I,C,Me	Me,AM,C
Cricetulus griseus	AAB86608.1	NA	NA	NA
Chelydra serpentina serpentina	AAN04908.1	NA	NA	NA
Apis mellifera	XP_624124.3	586-639	N,Z,C,Me	Me,TM,Z
Ixodes scapularis	XP_002400744.1	151-214	I,Co,Me	Me,TM,Z
Schistosoma mansoni	CAZ32701.1	504-569	Z,M,Co,Me	NB
Hydra magnipapillata	XP_002154415.1	500-563	M	Me,AM,C
Neohelice granulata	ACO59955.1	250-312	Z	Me,TM,Z
Paracentrotus lividus	ACN53783.1	612-671	C,M	Me,TM,Z
Strongylocentrotus purpuratus,	XP_790315.2	597-666	M,Z	Me,TM,Z
Saccoglossus kowalevskii,	XP_002741027.1	426-495	Z,Me	Me,AM,C
Branchiostoma floridae	XP_002613121.1	522-591	Z	Me,TM,Z
Narke japonica	BAA24230.1	560-629	I,Z	Me,AM,C
Takifugu rubripes,	O93279.1	598-667	I,Co,C,Me	Me,AM,C
Tetraodon fluviatilis	O73683.1	641-710	I	Me,AM,C
Danio rerio,	NP_690842.1	555-624	I,M	Me,TM,Co
Homo sapiens	P05067.3	631-700	I,C,Me	Me,AM,C

Calcium-binding	C
Iron-binding	I
Zinc-binding	Z
Metal-binding	Me
Nickel-binding	N
Magnesium-binding	M
Manganese-binding	Ma
Copper-binding	Co
Cobalt-binding	Cb
Transition Metal Binding	TM
Alkali Earth Metal Binding	AM
NonMetal Binding	NB
